# Should heart rate variability be “corrected” for heart rate? Biological, quantitative, and interpretive considerations

**DOI:** 10.1111/psyp.13287

**Published:** 2018-10-25

**Authors:** Eco J. C. de Geus, Peter J. Gianaros, Ryan C. Brindle, J. Richard Jennings, Gary G. Berntson

**Affiliations:** ^1^ Department of Biological Psychology Vrije Universiteit Amsterdam The Netherlands; ^2^ Departments of Psychology and Psychiatry University of Pittsburgh Pittsburgh Pennsylvania; ^3^ Department of Psychology & Neuroscience Program Washington and Lee University Lexington Virginia; ^4^ Department of Psychology The Ohio State University Columbus Ohio

**Keywords:** autonomic, behavioral medicine, heart rate, heart rate variability

## Abstract

Metrics of heart period variability are widely used in the behavioral and biomedical sciences, although somewhat confusingly labeled as heart rate variability (HRV). Despite their wide use, HRV metrics are usually analyzed and interpreted without reference to prevailing levels of cardiac chronotropic state (i.e., mean heart rate or mean heart period). This isolated treatment of HRV metrics is nontrivial. All HRV metrics routinely used in the literature exhibit a known and positive relationship with the mean duration of the interval between two beats (heart period): as the heart period increases, so does its variability. This raises the question of whether HRV metrics should be “corrected” for the mean heart period (or its inverse, the heart rate). Here, we outline biological, quantitative, and interpretive issues engendered by this question. We provide arguments that HRV is neither uniformly nor simply a surrogate for heart period. We also identify knowledge gaps that remain to be satisfactorily addressed with respect to assumptions underlying existing HRV correction approaches. In doing so, we aim to stimulate further progress toward the rigorous use and disciplined interpretation of HRV. We close with provisional guidance on HRV reporting that acknowledges the complex interplay between the mean and variability of the heart period.

## 1 INTRODUCTION

Heart rate variability (HRV) constitutes a parameter of physiology of long‐standing interest to behavioral and biomedical scientists. In the biomedical setting, HRV metrics are often used for risk stratification, where clinical end points (e.g., myocardial infarction) across a range of chronic health conditions may be forecasted by earlier measurements of HRV. Low HRV, for example, is associated with mortality in patients with coronary artery disease (Huikuri & Stein, 2013; Martin et al., 1987), chronic heart failure (Nolan et al., 1998), and among those with a history of myocardial infarction (Bigger, Fleiss, Rolnitzky, & Steinman, 1993; Bigger et al., 1988, 1992; Buccelletti et al., 2009; Camm et al., 2004; Kleiger, Miller, Bigger, & Moss, 1987b). Beyond mortality, hypertension (Singh et al., 1998), end‐stage renal disease (Brotman et al., 2010), and diabetes (Schroeder et al., 2005) are also associated with low HRV. Although such clinical associations may partly reflect impaired autonomic or vagal control caused by disease pathology, lowered HRV does not simply indicate disease severity, as it also predicts all‐cause mortality (Dekker et al., 1997; Zulfiqar, Jurivich, Gao, & Singer, 2010) and risk of cardiac morbidity and mortality (de Bruyne et al., 1999; Dekker et al., 2000; Hillebrand et al., 2013; Liao et al., 1997; Molgaard, Sorensen, & Bjerregaard, 1991; Tsuji et al., 1994) in apparently healthy subjects. The latter may be attributed in part to vagal inhibition of ventricular fibrillation (Schwartz, Billman, & Stone, 1984; Schwartz, La Rovere, & Vanoli, 1992; Schwartz et al., 1988). Notably, higher HRV does not always signal apparent protection, as high HRV confers risk for atrioventricular (AV) block, sick sinus syndrome, and atrial fibrillation (Fu, Huang, Piao, Lopatin, & Neubig, 2007; Vikman et al., 2003).

In addition to clinical applications, HRV metrics are often employed to better understand the peripheral physiological correlates of complex brain and behavioral processes, such as emotion and its regulation (Graziano & Derefinko, 2013; Rottenberg, Clift, Bolden, & Salomon, 2007) and executive cognitive functioning (Thayer & Lane, 2000; Thayer, Hansen, Saus‐Rose, & Johnsen, 2009), possibly by reflecting the functionality of higher brain systems, such as the prefrontal cortex (Beauchaine & Thayer, 2015). The main goal of the use of HRV metrics in these behavioral applications is to draw more specific inferences about autonomic nervous system (ANS) activity than are enabled by end‐organ metrics, such as the heart period. The long‐held perspective on the ANS as a “reciprocal system” reflected in the concept of sympathovagal balance is meaningful during strong manipulations like postural tilting and exercise (Goldberger, 1999), but this perspective has proven untenable in other contexts (Eckberg, 2000). It is now well established that the two arms of the ANS do not function reciprocally across many behavioral states and indeed may show coactivation in many contexts, including orienting reactions (Berntson, Cacioppo, & Quigley, 1991; Berntson, Cacioppo, Quigley, & Fabro, 1994; Gianaros & Quigley, 2001) or passive coping tasks (Bosch et al., 2001). Changes in heart period, therefore, cannot be necessarily interpreted as reflecting symmetric, but opposite, changes in cardiac sympathetic and parasympathetic (i.e., vagal) control. Instead, heart period and evoked changes in heart period are ambiguous with respect to their autonomic origins.

The autonomic space model provides a conceptual framework in which to understand reciprocal, independent, and coactive patterns of sympathetic and parasympathetic cardiac control, both in the context of within‐individual and between‐individual study designs (Berntson, Cacioppo, Binkley et al., 1994; Berntson, Cacioppo, & Quigley, 1994; Berntson, Norman, Hawkley, & Cacioppo, 2008). However, to be useful in empirical studies, the model requires separate measures of cardiac sympathetic and cardiac vagal activity. Although these measures could be obtained by pharmaceutical blockage of sympathetic and vagal activation, to do so is labor intensive, not without risk, hard to justify in children, and of limited practicality in larger‐scaled studies. Noninvasive metrics that predominantly capture either sympathetic or vagal activity are better suited for such studies. This has been a major driver for the development and use of HRV metrics in psychophysiology.

**BOX 1 What are the neurophysiological drivers of HRV?**

**Figure 1:**
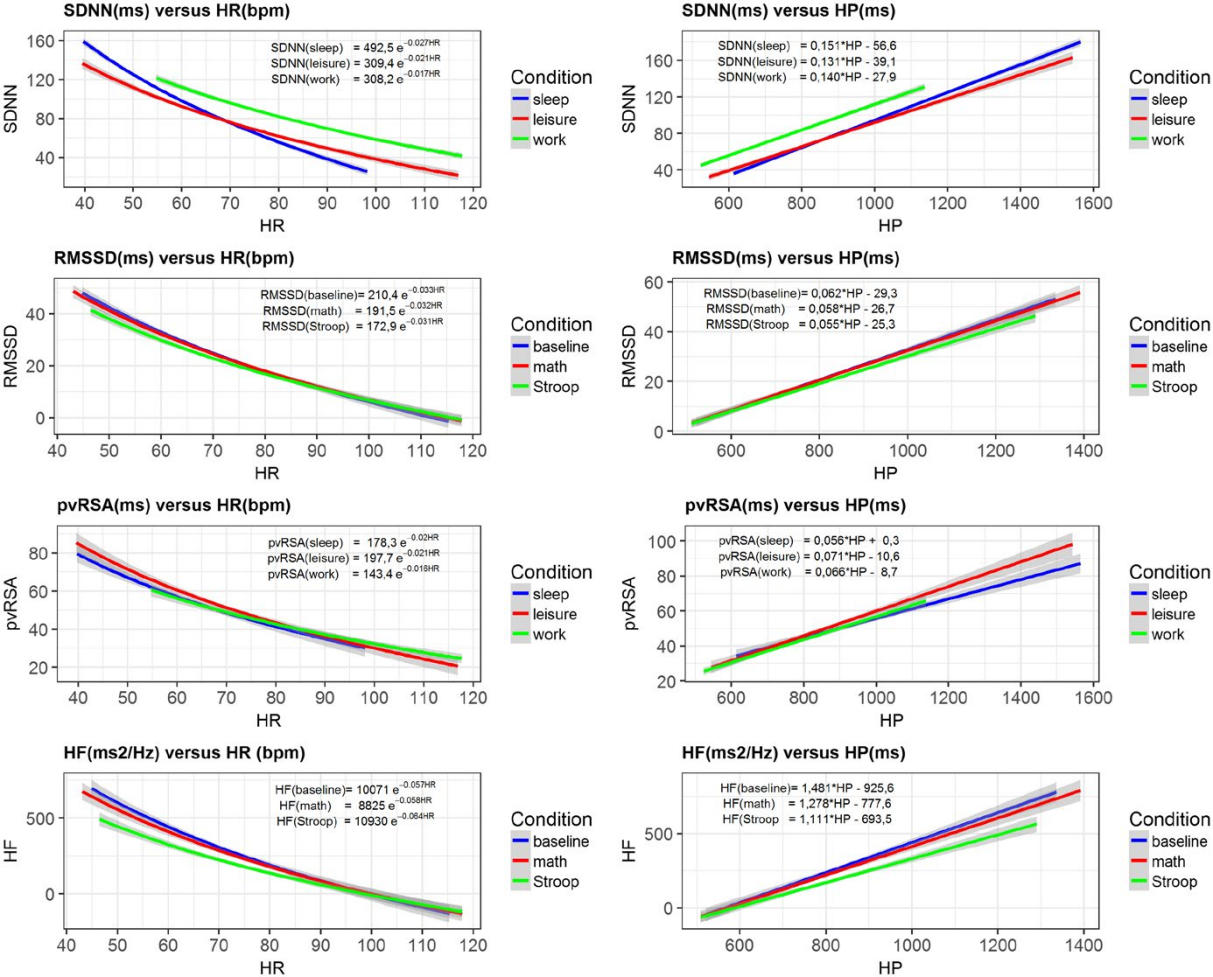
HRV metrics expressed as an exponential function of HR (bpm) and a linear function of IBI (ms). Data sources for SDNN and pvRSA are sleep (*N = *1,320), leisure time (*N = *1,277), and workday (*N = *958) averages obtained from ambulatory recordings on participants from the Netherlands Twin Register (NTR). Data sources for RMSSD and HF are the baseline (*N = *1,874), and math (*N = *1,778) and Stroop (*N = *1,794) condition averages from participants in the MIDUS II and Refresher Biomarker Studies. Left: Exponential fit (+ 95% CIs) of the HRV metrics against HR. Right: Linear fit (+ 95% CIs) of the HRV metrics against IBI

Both the parasympathetic and sympathetic arms of the ANS act on the cardiac pacemaker cells of the SA node. SA cells exhibit a special capacity for self‐excitation, which is characterized by spontaneous membrane depolarization and the consequent generation of rhythmic action potentials by the voltage clock and Ca^++^ mechanisms (Bartos, Grandi, & Ripplinger, 2015) that establish the intrinsic HR (HR in the absence of autonomic or hormonal influences). Several ion channels play a critical role in setting the rhythmic excitation of SA cells. Subsets of these ion channels are influenced by the release of acetylcholine (ACh) by the parasympathetic vagi onto muscarinic M2 receptors and by the release of norepinephrine (NE) by sympathetic motor neurons onto beta‐1 adrenergic receptors. ACh release strongly slows the spontaneous diastolic depolarization and may also increase the depth of repolarization of the SA cells (see Figure 1). This basic autonomic influence on SA activity leads to the well‐known observation that increases in the mean activity of the vagal nerve lead to increases in the heart period.

**Figure 2:**
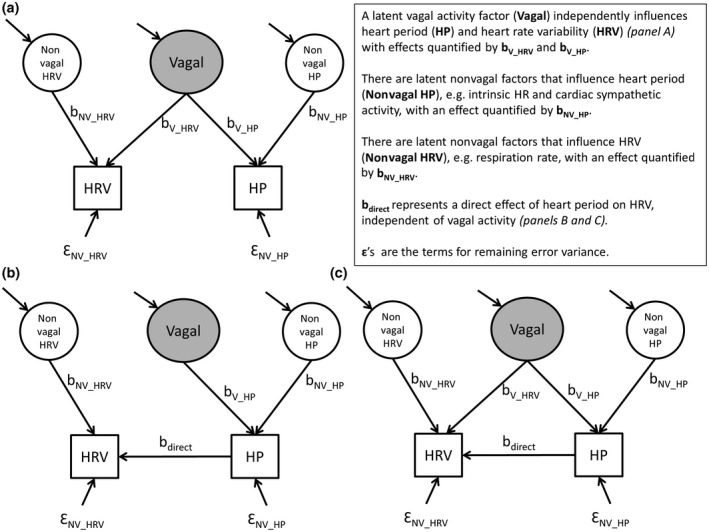
Models relating observable heart rate variability (HRV) and heart period to unobserved cardiac vagal activity

In parallel, increases in mean activity in the vagal nerve are accompanied by an increase in HRV through the principle of vagal gating (Eckberg, 1983, 2003). Vagal gating is based on two fundamental processes. First, tonic efferent vagal activity arising in the structures of the so‐called central autonomic network (Saper, 2002) is subject to phasic (frequency) modulation by other neurophysiological processes at the brain stem level, including cardiorespiratory coupling and the baroreflex (Berntson, Cacioppo, & Quigley, 1993). To elaborate, cardiorespiratory coupling exerts inhibitory influences during inspiration on vagal motor neurons in the nucleus ambiguus (NA), the predominant brainstem source of cardio‐inhibition by the vagal nerve in mammals (Chapleau & Abboud, 2001). This causes a periodic waxing and waning of the tonic vagal influence on SA node cells in phase with the respiratory cycle. This vagal influence translates into a decrease in heart period during inspiration relative to expiration, which is a chief source of high‐frequency HRV within normative rates of breathing. Figure 2a provides a schematic representation of this process.

We hasten to note that RSA neither implies a complete absence of vagal inhibition during expiration nor a complete vagal inhibition during inspiration,^1^ but rather relative changes in responsiveness of vagal motor neurons across the respiratory cycle, with less responsiveness during inspiration and more responsiveness during expiration. The ensuing modulation of central vagal activity by cardiorespiratory coupling and other neurophysiological sources of influence on RSA alter only a small fraction of the total influence of the vagus nerve on the SA node (Craft & Schwartz, 1995; Eckberg, 2003). For example, Craft and Schwartz performed full vagal blockade studies in 20 young (mean age 30) and 19 older (mean age 69) participants. In this study, the heart period shortened from 1,090 ms to 506 ms (**Δ**584 ms) in young participants, and from 1,053 ms to 718 ms (**Δ**335 ms) in older participants. These changes dwarf the typical modulation of heart period by phasic (respiratory‐related) inhibition that amounts to an average pvRSA of ~50 ms, with an approximate range of 0–200 ms.

It is critical to note here that respiratory influences also entrain sympathetic nervous system (SNS) outflow to SA node cells. The SNS outflow‐induced increase in NE release depolarizes and enhances the excitability of SA cells via metabotropic, cAMP‐mediated, second‐messenger processes. The latter processes not only accelerate the spontaneous depolarization of the SA cells, but also accelerate the speed of neural conduction in cardiac tissue. Compared to the fast (~400 ms) vagal influences, these sympathetic influences on HRV are strongly attenuated by the low‐pass filtering characteristics of slow (i.e., 2–3 s) G‐protein coupled metabotropic cascades that are initiated by NE binding at beta‐1 adrenergic receptors (Berntson et al., 1993; Mark & Herlitze, 2000). Thus, although both steady state and phasic increases in sympathetic SA node activity can shorten basal heart period, high frequency sympathetic fluctuations (e.g., in the respiratory frequency range) do not translate into phasic heart period fluctuations. Accordingly, most HRV metrics that are usually employed in psychophysiology and behavioral medicine (i.e., RMSSD, HF, pvRSA) can be largely ascribed to modulation of the vagal nerve outflow to SA cells.

A second fundamental principle in vagal gating is that the amplitude of the phasic modulation of activity in the autonomic motor neurons at the brainstem level (e.g., the NA) is a function of the absolute tonic level of firing of these autonomic motor neurons (Eckberg, 2003). The amplitude of the final modulated vagal signal traveling to the SA node therefore scales with the frequency of the tonic vagal pulse train presumptively arising in brain systems and cell groups comprising the so‐called central autonomic network. This means that the modulation of a pulse train of 12 Hz to vagal motor neurons will yield a larger peak‐to‐trough difference in the vagal signal to the SA node than the modulation of a 6 Hz pulse train. This is illustrated in Figure 2b. Here, we depict a person with lower centrally generated tonic vagal activity than in Figure 2a, which leads to a smaller difference between the shortest and longest beats in inspiration and expiration (50 ms compared to 100 ms). This principle is attributable to the fact that, at high levels of neural activity, there is a larger “carrier signal” to be subjected to phasic (respiratory‐related) inhibition.

As explained in detail in Box 1, specific measures of HRV, such as peak‐to‐valley respiratory sinus arrhythmia (pvRSA) and related metrics of heart period oscillations within common breathing frequencies, capture the inspiratory shortening and expiratory lengthening of heart periods across the respiratory cycle that is predominantly due to variations in cardiac vagal activity. In combination with measures that predominantly capture cardiac sympathetic activity, such as the pre‐ejection period (PEP), metrics of RSA may be interpreted and treated to meaningfully understand autonomic cardiac regulation within a two‐dimensional autonomic space model beyond ambiguous end‐organ activity provided by cardiac chronotropic metrics like heart period (Berntson, Cacioppo, Binkley et al., 1994; Bosch, de Geus, Veerman, Hoogstraten, & Nieuw Amerongen, 2003; Cacioppo et al., 1994). A 2007 special issue of *Biological Psychology* on cardiac vagal control illustrated its widespread use and highlighted issues pertaining to the use and abuse of various HRV metrics (Allen & Chambers, 2007). A recurrent concern has been the sometimes uncritical use of RSA as an index of vagal tone (see Box 2). In the decade since the publication of that special issue, interest in RSA and other HRV metrics has only expanded and deepened. This interest, however, has partly revived debate over a key and still open question addressed in this paper: Should HRV be “corrected” for heart rate (HR)? Based on a seminal paper by Monfredi and colleagues in 2014 (Monfredi et al., 2014), a rather strong viewpoint has been advocated that HRV is “just a nonlinear surrogate for HR” (Boyett, 2017; Boyett et al., 2017). Clearly, if HRV is confounded by a direct effect of the cardiac chronotropic state itself, this would fundamentally complicate its use to specifically capture one branch of the ANS.

**BOX 2 RSA and cardiac vagal activity**

The observation that RSA scales with levels of tonic vagal activity is the source of the widespread use of RSA as an index of vagal tone, a vague concept variably used to denote parasympathetic activity generated by the central autonomic network, the baroreflex circuitry, or simply the net effect of ACh on the SA node. However, inferring absolute levels of vagal activity at cortical, limbic, brainstem, or even SA node levels from any particular quantitative value of RSA is neither simple nor straightforward for many reasons. First, depth and rate of breathing strongly impact HRV metrics, especially those that index RSA (Eckberg, 2003; Grossman & Kollai, 1993; Grossman & Taylor, 2007; Grossman, Karemaker, & Wieling, 1991; Kollai & Mizsei, 1990; Taylor, Myers, Halliwill, Seidel, & Eckberg, 2001). Within individuals, RSA is inversely related to respiration rate and directly related to tidal volume. Hence, rapid and shallow breathing yields low RSA. The important observation here, which has been demonstrated many times over, is that an increase in RSA by slowing respiratory rate and increasing volume may be seen in the absence of any change in tonic vagal activity, as reflected in unchanged or even slightly decreasing mean heart period (Chapleau & Abboud, 2001). The impact of differences in breathing behavior on between‐individual comparisons of RSA is somewhat harder to gauge, but cannot be ignored. Given the importance of respiratory rate and tidal volume as critical determinants of RSA values independent of cardiac vagal activity, RSA measures are often obtained under controlled breathing conditions or they are statistically corrected for spontaneous variation within and between individuals, albeit with varying degrees of rigor (Ritz & Dahme, 2006).

A second reason not to equate RSA with tonic vagal activity is that the translation of fluctuations in vagal activity at the SA node into the actual slowing/speeding of the pacemaker potential is dependent on a complex interplay of postsynaptic signal transducers in the SA cells. Between‐individual differences and within‐individual changes in the efficiency of these transducers will distort any simple one‐to‐one mapping of vagal activity on HRV metrics. A classic example is the paradoxical reduction in HRV metrics at high levels of cardiac vagal activity induced in within‐individual designs by infusing pressor agents (Goldberger, Ahmed, Parker, & Kadish, 1994; Goldberger, Challapalli, Tung, Parker, & Kadish, 2001; Goldberger, Kim, Ahmed, & Kadish, 1996). Here, a saturation of a core element of postsynaptic ACh signal transduction, the SA muscarinic M2 receptors, causes low HRV in the presence of high vagal activity. A similar ceiling effect in the M2‐receptor signaling cascade may occur in regular vigorous exercisers with strong bradycardia. During nighttime, when their heart periods are much longer compared to daytime, these individuals exhibit a paradoxical lowering of RSA (van Lien et al., 2011).

Notwithstanding the many pitfalls highlighted thus far, RSA offers our best opportunity for estimating cardiac vagal activity noninvasively, most notably in larger‐scaled research in humans. We lack means for directly recording efferent vagal nerve activity to the heart, and pharmacological blockade suffers from its own disadvantages apart from being only feasible in small sample size studies. Various findings suggest that, in general, we can expect higher RSA with higher average levels of cardiac vagal activity. Within individuals, this is illustrated by gradual pharmacological blockade of ACh effects on the SA cells, which exerts no effects on respiratory behavior but is loyally tracked by parallel changes in RSA (Grossman & Taylor, 2007). Various studies have addressed this issue by administering a parasympathetic antagonist during a resting baseline condition and inferring vagal activity from the resultant decrease in heart period (Fouad, Tarazi, Ferrario, Fighaly, & Alicandri, 1984; Grossman & Kollai, 1993; Hayano et al., 1991; Kollai & Mizsei, 1990). RSA was estimated in parallel (e.g., with the peak‐to‐valley method). If pvRSA was completely proportional to cardiac vagal activity, then a perfect between‐individual correlation of the increases in heart period and pvRSA would have been observed. The actual correlations were quite appreciable but not perfect, even under controlled breathing conditions and incompletely saturated M2 receptors, varying between 0.5 and 0.9.

**BOX 3 A more in‐depth look at vagal stimulation studies**

**Figure 3:**
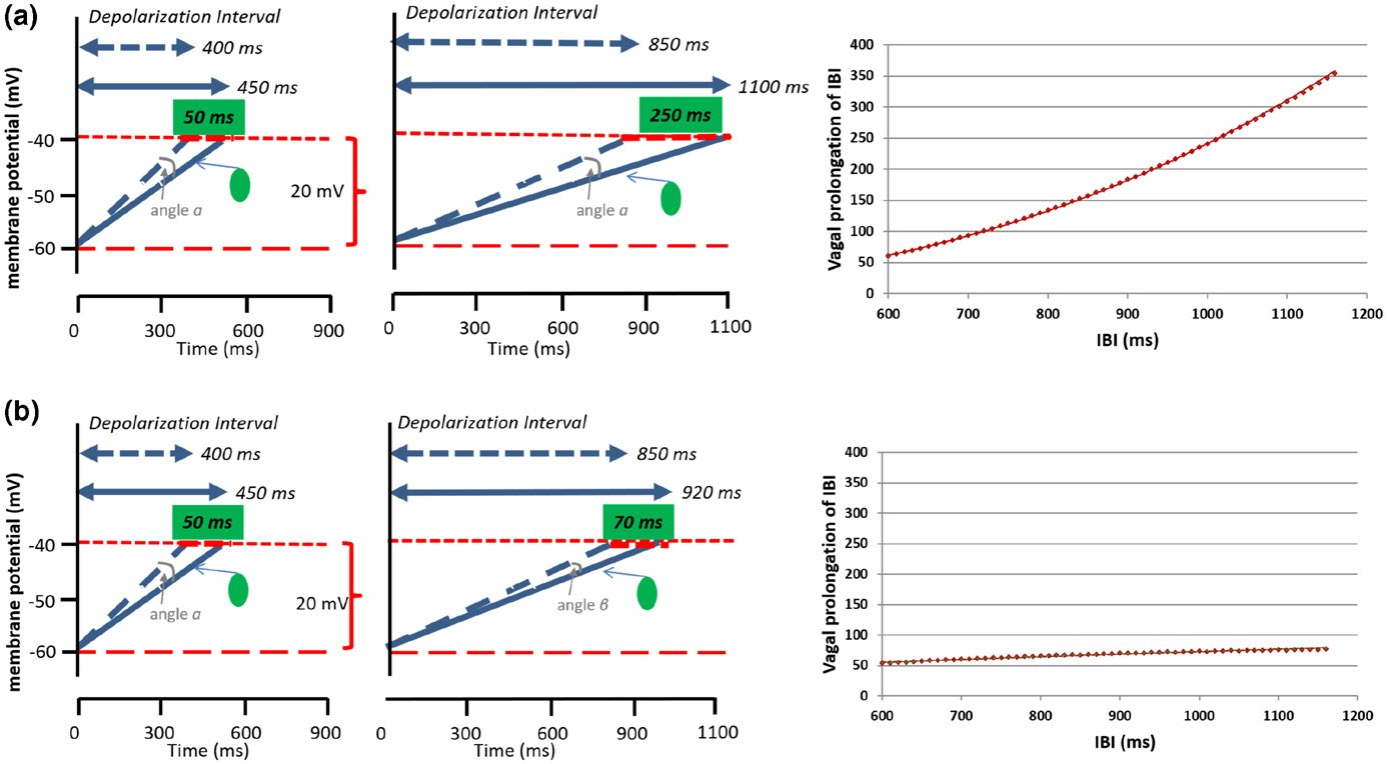
Effects of ACh release on the diastolic depolarization rate of the pacemaker cells in the SA. (a) Fixed angle scenario. The same amount of ACh release decreases the slope of diastolic depolarization by a fixed angle (α) at shorter (400 ms, left column) and longer (850 ms, middle column) diastolic depolarization intervals. This change prolongs the heart period less when the mean heart period is shorter (with faster mean diastolic depolarization of the pacemaker cells) than when mean heart period is longer (+50 ms vs. +250 ms). The graph on the right provides an illustration of this strong accumulative vagal prolongation effect across a heart period range of 600 to 1,200 ms. (b) Relative angle scenario. The same amount of ACh release decreases the slope of diastolic depolarization of the pacemaker cells by angles (α) or (β) that scale with the mean ongoing slope of diastolic depolarization. Hence, the effect on heart period is rather similar across shorter (400 ms, left column) and longer (850 ms, middle column) durations of the diastolic depolarization interval (+50 ms vs. +70 ms). The graph on the right provides an illustration of this weak vagal prolongation effect across a heart period range of 600 to 1,200 ms

Most of the vagal stimulation studies presented in Table 1 use a design in which the heart period attained during a steady state phase of vagal stimulation at a fixed frequency is compared to the heart period at prestimulation baseline. This procedure is repeated across a number of different vagal stimulation frequencies. The change in the heart period over the baseline heart period is computed for each frequency and, when plotted against stimulation frequency, typically yields a near‐perfect linear relation. The essence is that each stimulation event starts at the same baseline heart period, typically in the denervated heart (i.e., in the presence of bilateral vagal sectioning with sympathetic ganglia sectioning and/or sympathetic blockade). One could argue that this only indirectly answers the core question of dependency of vagal effects on the ongoing mean heart period. This potential limitation can be addressed by experimental manipulation of the baseline heart period before vagal stimulation commences, by changing cardiac sympathetic activity, or by changing nonautonomic effects on the diastolic depolarization rate, for example, by ivabradine or other blockers of the funny channel (decreasing I*f*). Testing the effects of vagal stimulation under different levels of concurrent cardiac sympathetic nerve stimulation (and hence baseline heart period) has been repeatedly done in the context of testing for accentuated antagonism (Quigley & Berntson, 1996). In mongrel dogs, the relative angle scenario in Figure 3b seemed to best fit the observed relationship between changes in vagal firing and chronotropic effects across different baseline values of heart period (Levy & Zieske, 1969a; Randall et al., 2003; Urthaler, Neely, Hageman, & Smith, 1986). When mean heart period levels were shortened by 30% to 35% through sympathetic stimulation at 4 Hz (S‐stim), a linear relation between vagal stimulation and heart period was again found in all studies, with comparable slopes between the S‐stim and no S‐stim conditions in two of the three studies (Table 1, lower). Combined manipulation of sympathetic and vagal tone by exercise in a conscious animal was used to manipulate basal mean heart period in another study (Stramba‐Badiale et al., 1991). When dogs (with a vagal stimulator) walked on a treadmill, their heart period changed from a resting value of 500 to 299 ms. In spite of this strong decrease in mean heart period, the slope obtained with vagal stimulation was comparable at rest (33.2 ms/Hz) and during exercise (28.8 ms/Hz).

We can conclude from these studies that, within a species, there is a relatively linear translation of phasic changes in vagal activity into changes in heart period across a wide range of baseline heart period levels with a reasonably stable slope. This is, again, what would have been predicted by the relative angle scenario in Figure 5b. However, in a dog model where central autonomic outflow was blocked, vagal pacing at 12 Hz produced lower increases in RSA when parallel sympathetic stimulation was applied (Hedman, Tahvanainen, Hartikainen, & Hakumaki, 1995). Pharmacological blockade in humans confirms that RSA is sensitive to moderate‐to‐large changes in cardiac sympathetic activity. As reviewed by Grossman and Taylor (2007), beta‐blockade in parallel increases heart period and RSA, even when vagal activity is not changed. This might be taken to suggest that there is indeed some direct effect of the mean heart period on RSA as would have been predicted by the fixed angle scenario in Figure 5a. Similarly, sympathetic agonists raising blood pressure like dobutamine cause a shortening of the mean heart period with a parallel decrease in HRV, when a baroreflex‐induced increase in vagal activity would be expected (Monfredi et al., 2014). Unfortunately, such effects on HRV could also occur independently of mediation by heart period, because sympathetic antagonists and agonists can interact directly with vagal activity at the brainstem level, and pre‐ and postjunctionally in the SA node (e.g., by the inhibitory action of the NE coreleased and the neuromodulator neuropeptide Y on ACh release (Quigley & Berntson, 1996).

A final class of relevant studies are those that used funny channel blockade to increase mean heart period (e.g., zetabradine or ivabradine). Funny channel blockade prolongs heart period, and many studies show that this bradycardia is coupled to a parallel increase in HRV (Borer & Le Heuzey, 2008; Kurtoglu et al., 2014). Vagal activity is still widely regarded to be the primary driver of HRV under ivabradine because atropine completely prevents the increase in HRV (Kozasa et al., 2018; Mangin et al., 1998). Nonetheless, the increase in HRV is counterintuitive, as ivabradine‐evoked bradycardia causes a parallel decrease in blood pressure. The latter causes a reflex increase in sympathetic nerve activity (Dias da Silva et al., 2015) and, one assumes, a reflex decrease in vagal activity in accord with baroreflex action. The observed increase in HRV was therefore explained as reflecting an “intrinsic dependency of HRV on pacemaker cycle length” (Dias da Silva at al., 2015, p. 32). This appears at first sight to be most compatible with the fixed angle scenario of Figure 5b.

However, using a murine genetic knockdown of HCN4, Kozasa et al. (2018) reported findings that were at odds with a fixed angle scenario. HCN4 is a main component of the funny channel, and this knockdown model mimics the bradycardic effects of ivabradine, as well as its positive, increasing effects on HRV. In the context of this model, they showed that funny channel action can directly impact the strength of vagal effects in the SA node. By counteracting K+ GIRK channels (reducing K+ efflux), the funny channel protects the SA cells against complete sinus pause under high vagal stimulation. Because the funny channel has a “limiter function” for the bradycardic effects induced by vagal activity, blocking it by ivabradine would act to amplify the effectiveness of phasic—for example, baroreflex or respiration‐induced—increases in vagal activity to induce phasic changes in heart period. The latter changes would serve to boost HRV. In keeping with this notion, amplifying funny channel action by HCN4 overexpression strongly reduced HRV, whereas mean heart period was unchanged. These results can all be explained by the funny channel counteracting the effectiveness of vagal activity without invoking an intrinsic dependency of HRV on heart period. Kozasa et al. (2018) also provide direct support for the relative angle scenario of Figure 5b. In isolated pacemaker cells, the basal diastolic depolarization rate in HCN4 knockdown mice was much slower than in the wild type animals (Kozasa et al., 2018, their figure D, p. 821). When exposed to increasing concentrations of ACh [0 to 30 nmol], the additional decrease in the depolarization rate induced by the same dose of ACh was much lower in the HCN4 knockdown (~35 mV/s) than in the wild type mice (~80 mV/s).

## 2 RELATIONSHIP BETWEEN HEART RATE, HEART PERIOD, AND HRV METRICS

A sometimes confusing use of nomenclature in the HRV literature merits careful consideration prior to raising the main issue of correcting HRV for HR. In practice, it is the variability in the time between heart beats—the interbeat interval (IBI) or the heart period in milliseconds—and not the HR in beats per minute that is the computational focus and source unit of measurement inherent to most HRV metrics. It is thus technically incorrect (or at least imprecise) to employ the term heart rate variability rather than heart period variability. Because the familiar abbreviation HRV actually already refers to heart period variability, we maintain this common usage. For the chronotropic state of the heart, however, heart period will be our preferred term rather than HR.

An obvious or at least intuitive reason to ask whether HRV should be corrected for HR is that all conventional metrics of HRV exhibit predictable relationships with prevailing (concurrent) levels of chronotropic state. These relationships have been appreciated for many decades, and they are evident both within and across individuals. Indeed, these relationships are evident with all standard HRV metrics derived from time and frequency domain analyses of human and nonhuman animal electrocardiogram data. Among others, these HRV metrics include those that capture the total variability of the heart period within an epoch (e.g., standard deviation of N‐N interval: SDNN), as well as the more popular root mean square of successive differences (RMSSD), spectral and autoregressive estimates of high‐frequency HRV (HF) and the canonical metric of RSA: pvRSA.^2^ When plotted against HR, the HRV metrics show a negative exponential relationship that is illustrated in two large human data sets in the left panels of Figure 1 (Dienberg Love, Seeman, Weinstein, & Ryff, 2010; Neijts et al., 2015; Sloan et al., 2017).

As can be seen in the right panels of Figure 1, the negative nonlinear relationship between HRV and HR predictably turns into a positive more linear one once we use intervals (in ms) versus rates (in bpm). This is because the conversion from rate to interval itself is a nonlinear inverse (i.e., HR = 60,000/heart period). Linearity is not perfect, however, and a power function often provides a slightly better fit between HRV and heart period (see online supporting information Figures S1 to S6 for complete data). Regardless, HRV metrics still exhibit a strong relationship with prevailing (concurrent) levels of cardiac chronotropic state—even when expressed as intervals (i.e., HRV tends to increase whenever heart period increases). The core problem that we address here is the nature of this relationship. Does it reflect a shared effect of autonomic (i.e., vagal) activity on both chronotropic state and its variability? Does it reflect a direct effect of chronotropic state on its variability? Do both of these effects coexist? Answers to these questions merit disciplined consideration by those using HRV across a range of basic and applied contexts, and they are critically relevant to the broader question of whether HRV should be corrected for HR across these contexts.

## 3 WHY ARE HRV AND HEART PERIOD CORRELATED?

From a neurophysiological perspective, a relationship between the prevailing chronotropic state—the heart period—and its variability is understandable (see Box 1). We reiterate essential points from Box 1 that higher tonic levels of vagal activity will act in parallel to.

1. increase HRV through the vagal gating mechanisms by respiratory and baroreflex input to vagal motor neurons, and
2. increase the mean heart period by slowing the spontaneous diastolic depolarization of the sinoatrial (SA) pacemaker cells.

Thus, the mean heart period is neurophysiologically “hard‐wired” to its variability. The idea is depicted in the model in Figure 2a. In this depiction, a latent variable of (centrally generated tonic) vagal activity independently influences both heart period and HRV. These, in turn, are influenced by other latent variables that include processes influencing (a) the intrinsic chronotropic state, (b) cardiac sympathetic activity, (c) respiratory activity, as well as (d) sensitivity to lung‐stretch reflexes, the baroreflex, and other phenomena impacting HRV.

If the model in Figure 2a is the true model, adjusting HRV for its observed relationship with the prevailing chronotropic state will lead to an underestimation of the association between vagal activity and HRV. This is quite different if the second model depicted in Figure 2b is the true model. Here, the heart period is the sole driver of HRV, which acts as “just a nonlinear surrogate for HR”—a viewpoint that has been advocated with vigor by some (Boyett, 2017; Boyett et al., 2017; Monfredi et al., 2014). According to the strong version of this latter viewpoint, no added value is provided by HRV over that contained in the prevailing heart period. Hence, HRV is a poorer marker of vagal activity than heart period itself because we just add noise from nonvagal sources. However, Model 4b is arguably incompatible with an explanation of HRV that is based on the neurophysiological coupling mechanisms discussed above. It could not, for instance, satisfactorily explain the clear uncoupling of RSA and heart period induced by respiratory manipulation. Such uncoupling counters the notion that changes in heart period are invariably a necessary condition (causal) for changes in RSA.

A final model to consider is the hybrid model depicted in Figure 2c with a latent variable influencing both mean heart period and HRV, but still allowing for some direct effect of heart period on HRV that is independent of vagal activity. If this model is correct, adjusting HRV for its observed relationship with heart period would still lead to an underestimation of its association with vagal activity. However, not correcting for heart period could lead HRV to overestimate vagal activity, with the severity of this imprecision depending on the effect size of the direct path. If this Model 4c is the true model, we would want to correct HRV only for the direct effect of heart period. The latter is nontrivial, as we typically do not know the values of b_V_HP_, b_V_HRV_ or b_direct_.

From the above, we deduce that the necessity to correct RSA and other HRV metrics for the heart period is closely tied to the core question of the presence and size of a direct effect of heart period on its variability. Put differently, apart from the understood neurophysiological link through the respiratory vagal gating outlined above, is there some intrinsic heteroscedasticity—a quantitative “dependency” of the variability in heart period on its mean? Or, even more simply, should we adopt the model in Figure 2c over that in Figure 2a?

## 4 IS HEART PERIOD A DRIVER OF HRV, INDEPENDENT OF VAGAL ACTIVITY?

For RSA, heteroscedasticity would be in play when the ongoing mean level of the heart period would determine the effect of phasic respiratory‐coupled changes in vagal activity on the difference in the longest and shortest heart period in a respiratory cycle. In such a scenario, a reduction in vagal activity from 12 to 10 spike trains per second during inspiration could lead to a phasic shortening of the heart period that would scale, for instance, linearly with the mean heart period. As a result, identical changes in vagal activity will yield a larger respiratory‐induced variance in heart period if the mean heart period is longer. A possible biophysical mechanism for a direct causal dependency of heart period variance on the mean heart period was proposed by Monfredi et al. (2014) based on the original work by Rocchetti et al. (Rocchetti, Malfatto, Lombardi, & Zaza, 2000). The proposed mechanism is nicely illustrated in an editorial by Stauss (see Stauss, 2014, figure 2, p. 1185), of which we capture the geometric essence in Figure 3a. This proposed mechanism critically assumes that the effects of ACh (acetylcholine) on the steepness of the diastolic depolarization rate of pacemaker cells are independent of the mean heart period (“I*_per_* will change the slope of the pacemaker potential by roughly the same amount regardless of rate,” Monfredi et al., 2014, p. 1339).

Based on simple geometric principles, phasic vagal effects on heart period will scale, in this “fixed angle” scenario, with the mean heart period. In the example provided in the upper panel of Figure 3, a change in one unit of vagal activity across a respiratory cycle leads to a change in diastolic depolarization duration of 50 ms at a mean heart period of 560 ms (taken an action potential duration of 160 ms). By contrast, at a mean heart period of 1,010 ms, the same change in vagal activity yields a much larger effect of 250 ms. The graph at left of Figure 3 shows the near‐linear prolongation of the heart period by vagal activity as a function of the mean heart period under a fixed angle scenario. Put simply: for the exact same phasic change in vagal activity, the induced variance in mean heart period captured by RSA (or other HRV metrics) is higher at longer mean heart periods than at shorter mean heart periods (i.e., strong heteroscedasticity).

Again, this fixed angle scenario builds on the critical assumption that the effects of ACh on the diastolic depolarization rate are independent of the currently ongoing diastolic depolarization rate. An alternative scenario for the effects of changes in vagal firing is the “relative angle” scenario in Figure 3b. Here, across most of the normal physiological heart period range, changes in vagal activity cause an absolute change in heart period that is nearly independent of its mean level. This scenario critically assumes that the effects of ACh on the diastolic depolarization rate of pacemaker cells scale as a function of that same rate. Hence, at slower diastolic depolarization rates and thus a longer mean heart period, further reductions in this rate induced by ACh are smaller than at faster diastolic depolarization rates with shorter mean heart periods. Apart from receptor binding kinetics, a major source for this scaling effect could be the increased opposition of osmotic drive on potassium ions by the electrostatic driving force, which is larger if repolarization/hyperpolarization is more profound. Also, slower diastolic depolarization rates allow more breakdown of ACh by ACh‐esterase (Dexter, Levy, & Rudy, 1989). In the relative angle scenario, a change of one unit vagal activity would, for instance, induce an absolute change in heart period of 50 ms when the mean heart period is 560 and an absolute change of 70 ms when the mean heart period is 1,010. Put simply: for the exact same phasic change in vagal activity, the induced variance in heart period captured by HRV metrics is nearly identical at longer and shorter mean heart periods (i.e., weak heteroscedasticity).

The above restates the original question: Is the phasic prolongation of the heart period by a fixed amount of vagal activity dependent on the mean heart period? as a new one: Is the phasic effect of a fixed amount of vagal activity on heart period dependent on the ongoing diastolic depolarization rate? To address this question, we first turn to studies that manipulated vagal activity by direct stimulation of the vagal nerve (Berntson, Quigley, Fabro, & Cacioppo, 1992; Carlson et al., 1992; de Neef, Versprille, Wise, & Jansen, 1983; Ford & McWilliam, 1986; Furukawa, Wallick, Carlson, & Martin, 1990; Levy & Zieske, 1969a, 1969b ; Parker, Celler, Potter, & McCloskey, 1984; Randall et al., 2003; Shimizu et al., 2009; Stramba‐Badiale et al., 1991; Urthaler et al., 1986). We start by noting that these stimulation procedures and associated findings again prove sensitive to the use of HR versus heart period as the chronotropic metric. When expressed as HR, for instance, the relation between vagal nerve firing rates and cardiac chronotropy is a nonlinear (hyperbolic) function (e.g., see classic study of Levy & Zieske, 1969a). However, as noted before (Quigley & Berntson, 1996), when expressed as heart period there is an approximately linear relation between the frequency of vagal stimulation and cardiac chronotropic state, and this linearity is a very robust finding across studies (see Table 1, upper). Moreover, an approximate linear relationship has also been reported between spontaneous variations in vagal activity and the ongoing heart period (Jewett, 1964; Katona, Poitras, Barnett, & Terry, 1970; Koizumi, Terui, & Kollai, 1985).

**Table 1:** Linear effects of vagal stimulation on heart period across species and conditions

Study	Species				Baseline IBI	Effect of right cardiac vagus stimulation on IBI (ms/Hz)	Vagal effect on IBI as % of baseline IBI	
							Study specific	Average for species
Carlson et al., 1992	Human				740	75	10.1%	10.1%
Furukawa et al., 1990	Dog				520	24.2	4.7%	
Levy & Zieske, 1969a	Dog				395	42	10.6%	
Levy & Zieske, 1969b	Dog				456	47.4	10.4%	
Parker et al., 1984	Dog				258	38	14.7%	
Randall et al., 2003	Dog				508	86.6	17.0%	
Stramba‐badiale et al., 1991	Dog				500	33.2	6.6%	
Urthaler et al., 1986	Dog				408	16.2	4.0%	11.4%
Berntson et al., 1992	Rat				240	7.4	3.1%	3.1%
de Neef et al., 1983	Rabbit				208	17.9	8.6%	
Ford & McWilliam, 1986	Rabbit				225	14.6	6.5%	
Shimizu et al., 2009	Rabbit				205	12.5	6.1%	7.5%
de Neef et al., 1983	Cat				256	27.8	10.9%	10.9%
de Neef et al., 1983	Guinea pig				218	26.5	12.2%	12.2%
These experiments repeated vagal stimulation without and with concurrent stimulation of the cardiac sympathetic nerve at 4 Hz:								
Levy & Zieske, 1969a		Mongrel dogs		without	395	42	10.6%	
Levy & Zieske, 1969a		Mongrel dogs		with	284	39.2	13.8%	
Randall et al., 2003		Mongrel dogs		without	508	86.6	17.0%	
Randall et al., 2003		Mongrel dogs		with	316	42.2	13.4%	
Urthaler et al., 1986		Beagle puppies		without	408	16.2	4.0%	
Urthaler et al., 1986		Beagle puppies		with	317	15.8	5.0%	
This experiment repeated vagal stimulation with the dogs standing quietly at the treadmill or forced to run until a heart rate of 200 bpm was reached:								
Stramba‐Badiale et al., 1991		Dog		Standing quiet	500	33.2	6.6%	
Stramba‐Badiale et al., 1991		Dog		Running (200 bpm)	299	28.8	9.6%	

Note

Upper half of the table depicts results of studies that use vagal stimulation to decrease heart period from its baseline level achieved at compete autonomic denervation The (mostly right) vagal nerve was stimulated at various frequencies, and the increases in heart period from a baseline heart period are regressed on the vagal firing frequency to obtain the slope, which can be expressed in absolute units (ms/Hz) or as a percentage of the basal heart period. Lower half of the table depicts results of studies that repeated vagal stimulation at different levels of baseline heart period level, which were induced by sympathetic stimulation or exercise. IBI = interbeat interval.

The relative linearity between vagal activity and heart period may be attributable, in part, to a negatively accelerating accumulation of ACh at SA synapses with increasing vagal activity, as well as a positively accelerating effect of synaptic ACh concentration on cardiac chronotropy (Dexter, Levy, & Rudy, 1989; Dexter, Saidel, Levy, & Rudy, 1989). Evidence from microdialysis work on sinoatrial ACh is generally consistent with this model (Shimizu et al., 2010; Zhan et al., 2013). Notably, while an approximate linearity has been observed between vagal activity and heart period across species, there are apparent species differences in the slope of these functions (see Table 1). These disparate values can be attributed in part to differences in surgical and anesthesia procedures, subspecies used (e.g., in dog), artificial ventilation versus spontaneous breathing, use of stellate ganglia dissection versus beta‐blockade to remove sympathetic activity, and the site and characteristics of electrical stimulation. However, across (sub)species, they also reflect the different basal heart period of these (sub)species in keeping with the exponential (0.249* BW^0.25^) relationship of basal heart period and body weight (Opthof, 2000).

When organized by reference to basal heart period levels, shorter basal heart period appears generally associated with flatter vagal activity‐chronotropic response slopes and longer basal heart period with steeper slopes (*r* = 0.72). Between species, differences in phasic prolongation of the heart period by a fixed amount of vagal activity therefore indeed seem to depend on the species differences in mean heart period. This would support the desire to correct for the relationship between chronotropic state and HRV metrics when comparing different species. Indeed, Monfredi and colleagues (Monfredi, Zhang, & Boyett, 2015) largely base their proposition that HRV is “just a nonlinear surrogate of HR” (they did not use heart period) largely on a comparison between species including human, rat, rabbit, and experimental preparations of the SA node in rat and rabbit. However, the most striking species difference in their data (Monfredi et al., 2014) was that the HR was lower and HRV metrics larger in humans than they were in the four animal models (figure 1, p. 1336). The covariation of HRV and HR levels between the four animal models is far less conclusive, if not absent. This raises concerns about motivating a uniform or universal correction of HRV for HR based on the observed covariation of mean HRV and HR levels across rather diverse species and the mixture of conscious animals and experimental SA cell preparations.

The universal linear scaling of the effects of vagal activity on heart period in Table 1 appears more congruent with the relative angle scenario than the fixed angle scenario in Figure 3. It suggests that, at any level of heart period, a fixed increase in vagal firing induces a (nearly) similar prolongation of the heart period, as in the scatterplot in Figure 3b. Some caution is needed as there are a number of caveats in interpreting vagal stimulation studies, and there are also empirical findings in support of Figure 3a. We critically review these nuances in more detail in Box 3. Nonetheless, a conservative summary is that the current evidence is more favorable to the relative angle scenario in Figure 3b. The idea that vagal activity has a direct neurophysiological influence on both heart period and HRV seems uncontested by the vast majority of evidence across species and experimental designs and preparations. The existence of an additional direct nonvagal related effect of mean heart period on HRV is less certain, but cannot currently be dismissed. Different dependencies of vagal‐induced changes in heart period on the mean heart period may arise when the mean heart period is governed by the different relative contributions of intrinsic HR, vagal activity, or sympathetic activity encountered in the typical (human) psychophysiological or behavioral medicine measurement context.

## 5 WHAT ARE THE MOST COMMON HRV CORRECTION APPROACHES, AND WHAT ARE THEIR IMPLICATIONS?

At this point, we cannot decisively determine whether the model in Figure 2a or in 4c is the true model for humans, and under what conditions. Correction for heart period would make sense only if Figure 2c is the applicable model in that specific context. We will therefore avoid the term correction because we are not yet sure there is anything wrong, in need of correction. Hereafter, we thus employ the term adjustment as the more appropriate term. We next review and compare commonly employed methods for adjusting HRV and demonstrate how they impact the HRV metrics used and potential inferences we can draw after their application.

**Figure 4:**
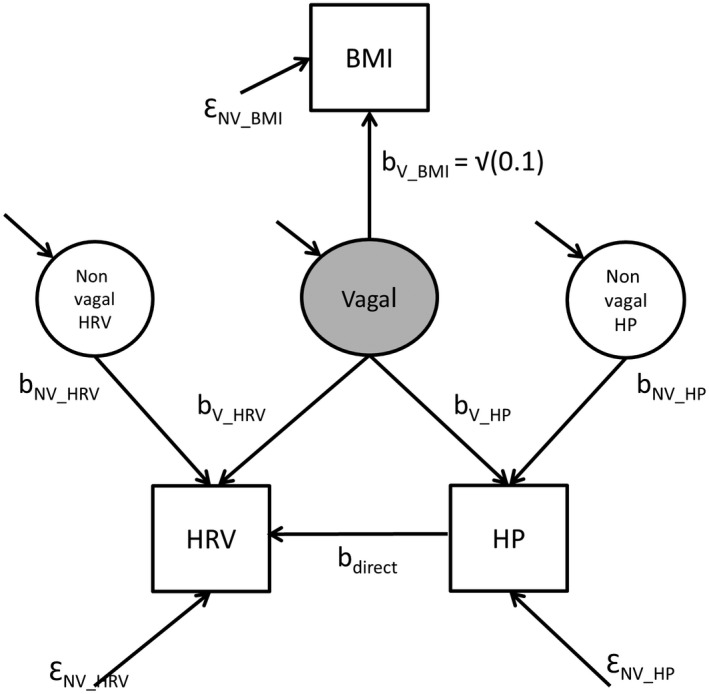
Structural equation model using HRV and heart period as observable indicators (facets) of a latent factor representing vagal nerve activity to test the association of vagal activity with BMI. Parameters b_V_BMI _and Ɛ_NV_BMI_ are set to values that cause vagal activity to explain 10% of the variance in BMI. As in Figure 2, b_V_HRV _and b_V_HP_ capture the vagal effects on HRV and heart period, and b_direct _the (putative) direct effect of heart period on HRV. Nonvagal (NV) and error (Ɛ) terms capture all other sources of variance in heart period and HRV

Most between‐individual applications of HRV use it as an indicator of a latent vagal activity construct that cannot be assessed directly. For instance, a research question could be whether vagal activity at baseline is associated with adiposity, as reflected by body mass index (BMI), as the outcome measure at follow‐up. We take BMI as a placeholder or illustrative outcome here, but the principle applies to any outcome (e.g., depressive symptoms, interleukin‐6 levels, hypertension, myocardial infarction, etc.). A first adjustment approach to account for the interrelationships between the prevailing heart period and its variability is to use linear regression analysis, either by using an HRV score adjusted by its covariance with heart period or by using HRV and heart period as simultaneous predictors. To illustrate what happens during adjustment by such an approach, we take the structural equation model in Figure 4 and use it to simulate data sets using the nine different parameter settings displayed in Table 2 (for details, see the R script in supporting information Appendix S1). These parameter settings vary the reliability of HRV and heart period as indicators of the latent vagal activity factor and also vary the b_direct_ path between heart period and HRV to reflect no effect of heart period on HRV, a small effect of heart period on HRV, or a moderate effect of heart period. In the simulated data obtained using these different parameter settings, we test the ability of linear regression to estimate the effects of HRV and heart period on BMI in (a) models that use either HRV or heart period alone, (b) a covariate model that first corrects BMI for heart period and regresses the residual BMI on HRV, and (c) a model that uses heart period and HRV as joint predictors of BMI.

**Table 2:** Effects of adjustment for heart period as a covariate or adding it as a second predictor on the association between HRV and BMI under various settings for the parameters in Figure 4

Parameter Setting 1, no direct effect (Figure 2a): Vagal activity influences BMI (b_V_BMI _= 0.316), IBI (b_V_IBI_ = 0.632), HRV (b_V_IBI_ = 0.632); no direct effect of IBI on HRV (b_direct_ = 0.0): IBI and HRV are equally influenced by vagal activity			
TRUE β_HRV_ & β_IBI_	Observed in regression analysis		
BMI = μ + 0.200*HRV	Model 1: HRV sole predictor	β_HRV _= 0.201	*R* ^2^: 0.044
BMI = μ + 0.200*IBI	Model 2: IBI sole predictor	β_IBI _= 0.203	*R* ^2^: 0.041
	Model 3: IBI as a covariate	β_HRV_ = 0.124	*R* ^2^: 0.015
	Model 4: HRV & IBI joint predictors	β_HRV_ = 0.148 β_IBI _= 0.150	*R* ^2^: 0.058
Parameter Setting 2, no direct effect (Figure 2a): Vagal activity influences BMI (b_V_BMI _= 0.316), IBI (b_V_IBI_ = 0.632), HRV (b_V_IBI_ = 0.316); no direct effect of IBI on HRV (b_direct_ = 0.0): HRV is more influenced by vagal activity than IBI			
TRUE β_HRV_ & β_IBI_	Observed in regression analysis		
BMI = μ + 0.200*HRV	Model 1: HRV sole predictor	β_HRV _= 0.196	*R* ^2^: 0.038
BMI = μ + 0.100*IBI	Model 2: IBI sole predictor	β_IBI _= 0.101	*R* ^2^: 0.010
	Model 3: IBI as a covariate	β_HRV_ = 0.176	*R* ^2^: 0.031
	Model 4: HRV & IBI joint predictors	β_HRV_ = 0.183 β_IBI _= 0.066	*R* ^2^: 0.042
Parameter Setting 3, no direct effect (Figure 2a): Vagal activity influences BMI (b_V_BMI _= 0.316), IBI (b_V_IBI_ = 0.316), HRV (b_V_IBI_ = 0. 632); no direct effect of IBI on HRV (b_direct_ = 0.0): IBI is more influenced by vagal activity than HRV			
TRUE β_HRV_ & β_IBI_	Observed in regression analysis		
BMI = μ + 0.100*HRV	Model 1: HRV sole predictor	β_HRV _= 0.095	*R* ^2^: 0.009
BMI = μ + 0.200*IBI	Model 2: IBI sole predictor	β_IBI _= 0.201	*R* ^2^: 0.040
	Model 3: IBI as a covariate	β_HRV_ = 0.055	*R* ^2^: 0.003
	Model 4: HRV & IBI joint predictors	β_HRV_ = 0.057 β_IBI _= 0.190	*R* ^2^: 0.043
Parameter Setting 4, small direct effect (Figure 2c): Vagal activity influences BMI (b_V_BMI _= 0.316), IBI (b_V_IBI_ = 0.632), HRV (b_V_IBI_ = 0.632); a small direct effect of IBI on HRV (b_direct_ = 0.1): IBI and HRV are equally influenced by vagal activity			
TRUE β_HRV_ & β_IBI_	Observed in regression analysis		
BMI = μ + 0.220*HRV	Model 1: HRV sole predictor	β_HRV _= 0.218	*R* ^2^: 0.047
BMI = μ + 0.200*IBI	Model 2: IBI sole predictor	β_IBI _= 0.199	*R* ^2^: 0.040
	Model 3: IBI as a covariate	β_HRV_ = 0.119	*R* ^2^: 0.015
	Model 4: HRV & IBI joint predictors	β_HRV_ = 0.158 β_IBI _= 0.121	*R* ^2^: 0.058
Parameter Setting 5, small direct effect (Figure 2c): Vagal activity influences BMI (b_V_BMI _= 0.316), IBI (b_V_IBI_ = 0.632), HRV (b_V_IBI_ = 0.316); a small direct effect of IBI on HRV (b_direct_ = 0.1): HRV is more influenced by vagal activity than IBI			
TRUE β_HRV_ & β_IBI_	Observed in regression analysis		
BMI = μ + 0.210*HRV	Model 1: HRV sole predictor	β_HRV _= 0.208	*R* ^2^: 0.043
BMI = μ + 0.100*IBI	Model 2: IBI sole predictor	β_IBI _= 0.097	*R* ^2^: 0.009
	Model 3: IBI as a covariate	β_HRV_ = 0.179	*R* ^2^: 0.032
	Model 4: HRV & IBI joint predictors	β_HRV_ = 0.196 β_IBI _= 0.038	*R* ^2^: 0.044
Parameter Setting 6, small direct effect (Figure 2c): Vagal activity influences BMI (b_V_BMI _= 0.316), IBI (b_V_IBI_ = 0.316), HRV (b_V_IBI_ = 0.632); a small direct effect of IBI on HRV (b_direct_ = 0.1): IBI is more influenced by vagal activity than HRV			
TRUE β_HRV_ & β_IBI_	Observed in regression analysis		
BMI = μ + 0.120*HRV	Model 1: HRV sole predictor	β_HRV _= 0.115	*R* ^2^: 0.013
BMI = μ + 0.200*IBI	Model 2: IBI sole predictor	β_IBI _= 0.196	*R* ^2^: 0.038
	Model 3: IBI as a covariate	β_HRV_ = 0.056	*R* ^2^: 0.003
	Model 4: HRV & IBI joint predictors	β_HRV_ = 0.062 β_IBI _= 0.178	*R* ^2^: 0.042
Parameter Setting 7, moderate direct effect (Figure 2c): Vagal activity influences BMI (b_V_BMI _= 0.316), IBI (b_V_IBI_ = 0.632), HRV (b_V_IBI_ = 0.632); a larger direct effect of IBI on HRV (b_direct_ = 0.3): IBI and HRV are equally influenced by vagal activity			
TRUE β_HRV_ & β_IBI_	Observed in regression analysis		
BMI = μ + 0.260*HRV	Model 1: HRV sole predictor	β_HRV _= 0.250	*R* ^2^: 0.062
BMI = μ + 0.200*IBI	Model 2: IBI sole predictor	β_IBI _= 0.192	*R* ^2^: 0.037
	Model 3: IBI as a covariate	β_HRV_ = 0.116	*R* ^2^: 0.014
	Model 4: HRV & IBI joint predictors	β_HRV_ = 0.227 β_IBI _= 0.032	*R* ^2^: 0.063
Parameter Setting 8, moderate direct effect (Figure 2c): Vagal activity influences BMI (b_V_BMI _= 0.316), IBI (b_V_IBI_ = 0.632), HRV (b_V_IBI_ = 0.316); a larger direct effect of IBI on HRV (b_direct_ = 0.3): HRV is more influenced by vagal activity than IBI			
TRUE β_HRV_ & β_IBI_	Observed in regression analysis		
BMI = μ + 0.230*HRV	Model 1: HRV sole predictor	β_HRV _= 0.222	*R* ^2^: 0.049
BMI = μ + 0.100*IBI	Model 2: IBI sole predictor	β_IBI _= 0.100	*R* ^2^: 0.010
	Model 3: IBI as a covariate	β_HRV_ = 0.152	*R* ^2^: 0.030
	Model 4: HRV & IBI joint predictors	β_HRV_ = 0.230 β_IBI _= −0.015	*R* ^2^: 0.049
Parameter Setting 9, moderate direct effect (Figure 2c): Vagal activity influences BMI (b_V_BMI _= 0.316), IBI (b_V_IBI_ = 0.632), HRV (b_V_IBI_ = 0.316); a larger direct effect of IBI on HRV (b_direct_ = 0.3): IBI is more influenced by vagal activity than HRV			
TRUE β_HRV_ & β_IBI_	Observed in regression analysis		
BMI = μ + 0.160*HRV	Model 1: HRV sole predictor	β_HRV _= 0.158	*R* ^2^: 0.025
BMI = μ + 0.200*IBI	Model 2: IBI sole predictor	β_IBI _= 0.193	*R* ^2^: 0.037
	Model 3: IBI as a covariate	β_HRV_ = 0.061	*R* ^2^: 0.004
	Model 4: HRV & IBI joint predictors	β_HRV_ = 0.153 β_IBI _= 0.042	*R* ^2^: 0.042

As shown in Table 2, regression models that use heart period and HRV as separate predictors recapture the contribution of HRV and heart period to BMI very well, independent of whether Figure 2a or 4c is the true data generating process. In all regression models, the combination of HRV and heart period yields the highest amount of variance explained in BMI, although, as may be expected from any semipartial correlation, the regression coefficients β_HRV_ and β_HP_ in the combined models underestimate the contribution of HRV and heart period compared to models using them as sole predictors. Importantly, a covariate analysis that first adjusts HRV for heart period will strongly underestimate the contribution of HRV to BMI, not just by underestimating the regression coefficient of HRV on BMI (β_HRV_), but also by a corresponding reduction in the amount of explained variance in BMI (*R* ^2^). The situation is most bleak in the parameter settings where heart period is a better indicator of vagal activity than HRV.

In real data, we of course only have access to the estimated β_HRV_ and β_HP_. Our interpretation of these estimates can be quite different depending on whether we expect Figure 2a or 4c to be the true data generating process. For instance, when we find the estimate β_HRV_ of 0.225 in the parameter settings #7 (generated under Figure 2c and a substantial direct effect of heart period on HRV), but believe the estimate to be generated under parameter settings #1 (generated under Figure 2a and a negligible direct effect of heart period on HRV), we would overestimate the contribution of HRV to BMI. Most dramatically, when we use HRV and heart period as dual predictors of BMI when the parameter settings #7 to #9 are true, but we instead assume the parameter settings #1 to #3 to be true, we would estimate the added predictive value of heart period to be small or even zero.

From these simulations, we conclude that adjustment for the HRV–heart period correlation through covariate adjustment is generally not to be recommended. Given the results in Table 2, it may not be surprising that studies that used a regression approach “competitively” combining heart period (or HR) with HRV metrics to test their relative prognostic importance for disease outcomes produced quite different conclusions (Abildstrom et al., 2003; Copie et al., 1996; Fleiss, Bigger, & Rolnitzky, 1992; Kleiger, Miller, Bigger, & Moss, 1987a; Tsuji et al., 1994). In most of the parameter settings in Table 2, adding heart period provides additional nonredundant information on cardiac vagal activity over that provided by HRV. Therefore, as has been suggested before, using both HRV and heart period as indicators of cardiac vagal activity may be the wisest course of action (Grossman & Taylor, 2007), but we should then interpret the *R* ^2^ as the best measure of explained variance by vagal activity and restrain from interpreting the magnitude of β_HRV_ and β_HP _separately (e.g., as signaling that either HRV or heart period are the better predictors.).

A second adjustment approach is to create a new “corrected” HRV measure that expresses HRV as a function of the mean heart period (or HR). We discuss two methods for such corrected HRV measures, although many more have been suggested (Sacha & Pluta, 2005; Sacha et al., 2013). A general procedure to create an HRV metric that is independent of chronotropic state was proposed by Monfredi et al. (2014) based on the exponential relationship between HRV metrics and HR. The core idea is to compare the HRV between individuals only after recasting it to a fixed reference HR. The procedure exploits the linear relationship between HR and the natural logarithm transformation of the HRV (LnHRV). A “corrected (c)” HRV (cHRV) is computed as the HRV that would have been obtained at a fixed reference HR. The latter is computed by transforming the observed HRV to LnHRV and then subtracting/adding the change in LnHRV that would occur if HR changed from the observed HR to the reference HR. This change can be derived from the slope of the linear relation between LnHRV and HR. If LnHRV = intercept + β*HR, then

(1) $$\left({\text{LnHRV}}_{\text{reference}}-{\;\text{LnHRV}}_{\text{observed}}\right)=\beta *\left({\text{HR}}_{\text{reference}}-{\;\text{HR}}_{\text{observed}}\right)$$

which, after exponentiation of both sides of the equation and some further algebra, gives

(2) $${\text{HRV}}_{\text{reference}}/{\text{HRV}}_{\text{observed}}=e^{\;\beta *(\text{HRreference}\;-\;\text{HRobserved})}$$

Based on their empirical data, Monfredi et al. (2014) estimate the slope (β) between LnSDNN and HR to be −0.017 (−1/58.8), yielding

(3) $${\text{SDNN}}_{\text{reference}}={\;\text{SDNN}}_{\text{observed}}/e{\;}^{\left(\left(\text{HRreference}\;-\;\text{HRobserved}\right)/58.8\right)}$$

Notwithstanding a reasonable comparability across different samples, the slope of LnSDNN and HR appears to be age dependent (van den Berg et al., 2018). It is also sensitive to the condition/situation in which HRV is measured. The latter is illustrated in the Netherlands Twin Register (NTR) and (Midlife in the United States) MIDUS data depicted in Figure 1. This suggests that the correction formula cannot use a single fixed slope of −0.017, but needs this slope to be based on the specific data set and measurement conditions of interest. We computed the “corrected” SDNN (cSDNN) as well as the corrected version for the other HRV metrics in the NTR and MIDUS samples using the condition specific slopes (see Table 3, 2nd column, e.g., cRMSSD during leisure time in NTR = RMSSD * e^−0,025*HR^). We then correlated the cHRV metrics with HR and the untransformed HRV. We first note that before adjustment pvRSA and HF measures are less strongly correlated with HR than SDNN and RMSSD. Second, the original relationship between HR and all four HRV metrics is completely removed by the Monfredi method. Third, the HR‐adjusted cHRV values preserve the rank ordering of individuals quite well, as shown by the high correlations between adjusted and unadjusted values.

**Table 3:** Effects of adjustment of the HRV metric by the approach proposed by Monfredi et al. (2014) on its correlation to HR, the unadjusted HRV, BMI, and age

	Slope of exponential relationship between HRV metric and HR	Correlation of HRV metric to HR before adjustment	Correlation of HRV metric to HR after adjustment	Correlation between unadjusted HRV and adjusted HRV metric	Correlation between BMI and HRV metric before adjustment	Correlation between BMI and HRV metric after adjustment	Correlation between age and HRV metric before adjustment	Correlation between age and HRV metric after adjustment
NTR								
SDNN								
During sleep	–0.027	–0.69	0.02	0.68	−0.15	−0.08	−0.35	−0.42
During work	–0.021	–0.52	0.02	0.82	−0.15	–0.18	−0.31	−0.45
During leisure time	–0.017	–0.62	0.02	0.75	−0.22	−0.26	–0.30	−0.49
RMSSD								
During sleep	–0.039	–0.55	0.04	0.76	–0.12	–0.06	–0.35	–0.37
During work	–0.035	–0.53	0.03	0.79	–0.14	–0.17	–0.29	–0.42
During leisure time	–0.025	–0.52	0.02	0.81	–0.18	–0.20	–0.21	–0.33
RSA								
During sleep	–0.020	–0.30	0.03	0.92	–0.08	–0.03	–0.37	–0.37
During work	–0.021	–0.36	0.03	0.88	–0.18	–0.20	–0.34	–0.42
During leisure time	–0.016	–0.39	0.02	0.89	–0.26	–0.29	–0.41	–0.49
MIDUS								
SDNN								
During baseline	–0.020	–0.40	0.01	0.89	–0.08	–0.06	–0.29	–0.39
During math	–0.019	–0.39	0.02	0.89	–0.11	–0.12	–0.27	–0.37
During Stroop	–0.017	–0.36	–0.01	0.91	–0.12	–0.13	–0.23	–0.31
RMSSD								
During baseline	–0.032	–0.44	–0.01	0.85	0.03	0.05	–0.18	–0.27
During math	–0.032	–0.47	–0.03	0.85	0.01	0.01	–0.18	–0.32
During Stroop	–0.031	–0.45	–0.03	0.86	0.00	–0.01	–0.15	–0.24
HF								
During baseline	–0.057	–0.24	–0.02	0.88	0.05	0.05	–0.15	–0.23
During math	–0.058	–0.29	–0.02	0.86	0.01	–0.01	–0.13	–0.24
During Stroop	–0.064	–0.26	–0.02	0.82	0.00	–0.02	–0.07	–0.14

Note

Mean age was 34.1 (± 9.6) in NTR and 54.7 (± 12.3) in MIDUS. Mean BMI was 24.0 (± 4.1) in NTR and 30.0 (± 7.0) in MIDUS.

A second method to create an HRV measure that is adjusted for chronotropic state uses the coefficient of variation (CV) of the HRV metrics, which for SDNN has the form

(4) $$cvSDNN=100*\frac{SDNN}{IBI}$$

but can be generalized to other HRV metrics as follows:

(5) $$cvRMSSD=100*\frac{RMSSD}{IBI}$$

(6) $$cvpvRSA=100*\frac{longest\;IBI\;during\;expiration-shortest\;IBI\;during\;inspiration}{mean\;IBI\;during\;breath}$$

(7) $$cvHF=100*\frac{HF}{{\left(IBI\right)}^{2}}$$

The more parsimonious and easy to compute cvSDNN metric was shown to closely approximate the cSDNN obtained by the adjustment proposed by Monfredi and colleagues (van Roon, Snieder, Lefrandt, de Geus, & Riese, 2016). After log transformation, the adjusted SDNN values from each transformation were highly correlated and only differed by a constant. We applied this CV adjustment of van Roon et al. (2016) to the HRV metrics in the MIDUS and NTR samples and correlated the cvHRV metrics with heart period and the untransformed HRV (see Table 4). A number of differences between the van Roon and the Monfredi adjustment methods are seen. First, the relationship between heart period and HRV metrics is attenuated, but not entirely removed, by the CV transformation. Second, the adjusted HRV values preserve the rank ordering of individuals on HRV almost perfectly, as shown by the very high correlations between unadjusted and adjusted values.

**Table 4:** Effects of adjustment of the HRV metric by the approach proposed by van Roon et al. (2016) on its correlation to heart period, the unadjusted HRV, BMI, and age

	Correlation of HRV metric to heart period before adjustment	Correlation of HRV metric to heart period after adjustment	Correlation between unadjusted HRV and adjusted HRV metric	Correlation between BMI and HRV metric before adjustment	Correlation between BMI and HRV metric after adjustment	Correlation between age and HRV metric before adjustment	Correlation between age and HRV metric after adjustment
NTR							
SDNN							
During sleep	0.58	0.36	0.90	–0.15	–0.12	–0.35	–0.41
During work	0.57	0.37	0.92	–0.15	–0.17	–0.31	–0.42
During leisure time	0.55	0.31	0.87	–0.22	–0.26	–0.30	–0.45
RMSSD							
During sleep	0.73	0.37	0.96	–0.12	–0.10	–0.35	–0.37
During work	0.59	0.25	0.97	–0.14	–0.15	–0.29	–0.35
During leisure time	0.67	0.24	0.95	–0.18	–0.19	–0.21	–0.27
RSA							
During sleep	0.30	0.03	0.95	–0.08	–0.04	–0.37	–0.37
During work	0.36	0.10	0.96	–0.18	–0.20	–0.34	–0.39
During leisure time	0.43	0.10	0.94	–0.26	–0.28	–0.41	–0.48
MIDUS							
SDNN							
During baseline	0.44	0.27	0.95	–0.08	–0.07	–0.29	–0.37
During math	0.47	0.30	0.95	–0.11	–0.12	–0.27	–0.35
During Stroop	0.45	0.29	0.95	–0.12	–0.13	–0.23	–0.30
RMSSD							
During baseline	0.39	0.11	0.97	0.03	0.04	–0.18	–0.23
During math	0.38	0.10	0.98	0.01	0.01	–0.18	–0.24
During Stroop	0.36	0.08	0.97	0.00	–0.01	–0.15	–0.19
HF							
During baseline	0.25	0.20	0.99	0.05	0.05	–0.15	–0.17
During math	0.30	0.24	0.99	0.01	0.00	–0.13	–0.16
During Stroop	0.27	0.23	0.99	0.00	0.00	–0.07	–0.09

We next investigate how these two adjustment methods affect typical between‐ and within‐individual designs used in the behavioral sciences. In the rightmost part of Tables 3 and 4, we correlate the unadjusted and adjusted HRV measures to the individuals’ BMI (known small effect size) and age (known moderate effect size). It can be easily observed that the adjustment creates no alarming discrepancies with results obtained using the unadjusted HRV for BMI. In contrast, the correlation with age of both the Monfredi cHRV metrics and the van Roon adjusted cvHRV metrics is systematically higher than for the unadjusted HRV metrics (confirmed by Fisher *Z* transform of the correlations). In short, the impact of adjustment on between‐individual designs may not be very large when the correlations between HRV and the predicted variables are small (like BMI), but for moderate correlations (like age) meaningfully different results may arise for adjusted and unadjusted HRV metrics.

The impact of the HRV adjustments on within‐individual study designs was more profound. This is illustrated in Table 5, where we exploit the repeated measures structure of HRV data from MIDUS (lab‐based within‐individual observations) and the NTR (ambulatory within‐individual values across a 24‐hr recording). Inspection of the changes in HRV over the baseline condition expressed in standard deviations of the baseline level (as a measure of the effect size) demonstrates that adjusting HRV for heart period can substantially influence the observed effects of (a) acute psychological stress on “vagal withdrawal,” as assessed by HRV suppression, and (b) expected decreases in vagal activity from sleep to leisure‐time activities and from leisure‐time to work‐related activities. The general pattern is that the adjusted HRV measures underestimate the effect sizes of the unadjusted HRV measures when heart period changes are modest. Moreover, contradictory conclusions can result from adjusted and unadjusted measures if heart period changes are larger. This is especially so for the Monfredi adjustment.

**Table 5:** Impact of HRV adjustment on the effect sizes in repeated measures analyses

	**Mean**	***SD***	**Mean**	***SD***	**NTR**							
	**HR**		**Heart period**									
Sleep	63.2	8.3	979.9	134.3								
Leisure	70.8	10.0	878.9	128.2								
Work	84.9	10.7	747.7	103.2	**Mean**	***SD***	**Δ (from sleep level)**			**Effect size**		
	**SDNN**		**cSDNN**		**cvSDNN**		**SDNN**	**cSDNN**	**cvSDNN**	**SDNN**	**cSDNN**	**cvSDNN**
Sleep	91.8	28.0	503.8	114.6	9.3	2.1						
Leisure	76.0	28.4	324.6	103.7	8.6	2.7	–15.7	–180.5	–0.71	–0.56	–1.57	–0.33
Work	76.6	21.4	317.0	69.7	10.2	2.2	–16.3	–191.9	+0.81	–0.58	–1.67	+0.38
	**RMSSD**		**cRMSSD**		**cvRMSSD**		**RMSSD**	**cRMSSD**	**cvRMSSD**	**RMSSD**	**cRMSSD**	**cvRMSSD**
Sleep	52.9	26.5	604.3	263.6	5.3	2.3						
Leisure	45.5	25.6	497.4	241.6	5.0	2.5	–7.3	–110.7	–0.24	–0.28	–0.42	–0.10
Work	32.8	15.6	257.0	104.5	4.3	1.8	–20.9	–355.9	–1.06	–0.79	–1.35	–0.45
	**pvRSA**		**cpvRSA**		**cvpvRSA**		**pvRSA**	**cpvRSA**	**cvpvRSA**	**pvRSA**	**cpvRSA**	**cvpvRSA**
Sleep	54.8	24.7	195.1	85.2	5.6	2.4						
Leisure	51.5	24.9	218.9	99.8	5.8	2.6	–3.3	+23.4	+0.23	–0.13	+0.27	+0.10
Work	40.3	15.8	153.4	55.7	5.4	1.9	–14.5	–41.6	–0.22	–0.59	–0.49	–0.09

	**Mean**	***SD***	**Mean**	***SD***	**MIDUS**							
	**HR**		**Heart period**									
Baseline	72.2	10.8	850.5	129.9								
Math	75.4	11.2	814.2	124.5								
Stroop	76.3	11.4	804.2	123.3	**Mean**	***SD***	**Δ (from baseline level)**			**Effect size**		
	**SDNN**		**cSDNN**		**cvSDNN**		**SDNN**	**cSDNN**	**cvSDNN**	**SDNN**	**cSDNN**	**cvSDNN**
Baseline	36.2	17.8	145.0	65.6	4.2	1.9						
Math	30.6	15.0	125.5	56.0	3.7	1.7	–5.4	–24.3	–0.49	–0.31	–0.37	–0.26
Stroop	29.8	15.6	107.1	50.6	3.7	1.7	–6.2	–42.6	–0.53	–0.35	–0.65	–0.28
	**RMSSD**		**cRMSSD**		**cvRMSSD**		**RMSSD**	**cRMSSD**	**cvRMSSD**	**RMSSD**	**cRMSSD**	**cvRMSSD**
Baseline	23.8	17.9	225.7	148.9	2.7	1.9						
Math	21.4	15.8	224.0	133.1	2.6	1.7	–2.4	–3.3	–0.18	–0.13	–0.02	–0.10
Stroop	19.5	15.1	194.0	128.7	2.4	1.6	–4.3	–32.9	–0.38	–0.24	–0.22	–0.20
	**HF**		**cHF**		**cvHF**		**HF**	**cHF**	**cvHF**	**HF**	**cHF**	**cvHF**
Baseline	344.4	760.4	17,607	32,784	38.1	79.8						
Math	269.0	525.5	17,406	27,536	30.9	56.9	–68.8	137	–6.50	–0.09	+0.001	–0.08
Stroop	203.7	500.3	20,903	42,557	23.5	54.4	–130.8	3,423	–13.68	–0.17	+0.10	–0.17

Note

**Δ** denotes the within‐individual change from sleep level (NTR) or baseline level (MIDUS). It can differ slightly from the subtraction of the condition means, because only subjects with complete data were used for the change scores. Effect size denotes the average size of the within‐individual change expressed as proportion of the standard deviation during the sleep (NTR) or baseline condition. c[HRV] adjustment by Monfredi method; cv[HRV] adjustment by van Roon method. Unadjusted effect sizes, effect size on c[HRV] adjustment, effect size on cv[HRV] adjustment.

The above serves as a demonstration that adjustment for heart period (or HR) is not interpretively neutral: it can profoundly affect the conclusions drawn about correlations to external variables and the effects of acute behavioral states (e.g., psychological stress) on cardiac autonomic activity or the variation in cardiac autonomic activity across a 24‐hr naturalistic recording. Pending resolution of the core question of the existence of a direct effect of heart period on HRV, we cannot tell which of these adjustment methods best captures the ground truth, or whether any adjustment is needed at all.

## 6 PROVISIONAL RECOMMENDATIONS

In view of the preceding biological, quantitative, and interpretive considerations, we close by offering several provisional recommendations on the handling and reporting of HRV metrics. First, when attempting to quantify cardiac vagal activity noninvasively, **we advocate the use of pvRSA** **and HF**, as they are based on the well‐understood neurophysiological mechanism of respiratory gating of vagal activity. The metric of RMSSD is also widely used, and it is highly correlated with pvRSA and HF (Goedhart, van der Sluis, Houtveen, Willemsen, & de Geus, 2007; Grossman, van Beek, & Wientjes, 1990). The former, however, does have somewhat different transfer functions across the respiratory frequency band, which are dependent on basal heart period (Berntson, Lozano, & Chen, 2005). Nevertheless, RMSSD has been well validated as a metric of HRV and may be preferable with records having ectopic beats that reset the cardiac rhythm.

With regard to adjustment for heart period, our second recommendation is to **always formally examine and report the relationship(s) between the prevailing heart period and the primary HRV metrics** under study. Depending on study designs and data sampling structures, such relationships should be tested within or across individuals and within or across conditions. “Full disclosure” type figures like Figures S1 to S6 are informative and could be routinely provided with publications as data supplements. At the very least, we recommend the reporting of zero‐order, and when relevant, partial correlations. Inherent to this recommendation is the corollary recommendation to routinely report descriptive statistics for mean or prevailing heart period (and optionally HR) values in each condition and relevant subgroup alongside reports of HRV metrics. Collectively, following these suggestions will strengthen transparency in reporting, and they may provide for cumulative insights into the magnitude of observable HRV–heart period dependencies across a range of empirical contexts in future research.

A third recommendation is to test and **report on how adjusting HRV metrics for chronotropic** **state affects primary study outcomes**, whether it is a cross‐sectional correlation analysis across individuals or an analysis of how an experimental intervention alters some (adjusted) HRV parameter. Hence, a conservative approach is to report main effects and associations of interest in both adjusted and unadjusted metrics. As a fourth recommendation, **we favor the parsimonious CV transformation over all other types of adjusted HRV measures**. It does not depend on a condition and population‐specific slope, such as the Monfredi adjustment. In keeping with our first recommendation, the cvpvRSA and cvHF would be our measures of first choice. Fifth, we recommend to **avoid adjustment approaches that involve “regressing out” heart period effects in covariate analyses **and assessing HRV in terms of residuals.

Any adjustment of HRV metrics for prevailing chronotropic states measured in terms of heart period still leaves intact the prior recommendations regarding the control for other sources of influence on HRV (e.g., respiratory rate, depth, cycle time effects). For example, the proposed adjustment for heart period is compatible (and can be done) with simultaneous correction for respiratory behaviors. A review of the optimal adjustment strategies in that realm is outside our scope, but we note that some of the issues introduced in the analyses in Figure 2 may also apply to dual correction for tidal volume and respiration rate (Ritz & Dahme, 2006) if these parameters, which are highly correlated under physiological conditions, reflect a shared latent generator of respiratory‐frequency autonomic rhythms (Eckberg, 2003).

A sixth recommendation is to **avoid mixing units of measurement **(e.g., ms vs. bpm; ms vs. spectral density units) when examining HRV and chronotropic state relationships or adjusting for them. Failure to do so can have serious consequences that will not only mislead readers, but also undermine inferences. Although less familiar to the broader readership, using heart period in the context of HRV is more appropriate than using HR. Any transformations, including transformation to normality, should be done on the final metrics, not in between. For instance, computing the coefficient of variation of HF spectral power by dividing the (natural) log‐transformed HF power in ms^2^ by HR in bpm is a recipe for double trouble: mixing (a) units and (b) transformed and untransformed values.

A final recommendation is to **be explicit about the theoretical model for the relationships between vagal activity, heart period, and HRV** and to use an analytic strategy that is in line with that model. This can be taken as a plea for approaches, such as structural equation models of the type shown in Figure 4. Such models can be readily expanded by a latent variable for cardiac sympathetic activity that can be indexed by the PEP. Moreover, they can additionally accommodate indicators of nonvagal effects, like respiration rate and depth, and even, provided it is known from blockade, the intrinsic HR.

## 7 WHERE DO WE GO FROM HERE?

At present, there are no easy answers to the question of whether HRV should be corrected for heart rate. It is not even clear that anything is in need of correction per se, leading to our preferred term, adjustment. In this regard, we maintain that there are many knowledge gaps in our understanding of the meaning of HRV metrics that have undergone adjustment. We specifically caution that adjustment approaches may in fact remove meaningful variance in outcomes of interest that can be attributable to autonomic and neurophysiological phenomena. If an adjustment for cardiac chronotropic state (heart period or rate) is employed, it is incumbent on the author(s) to justify the specific adjustment within the given context. We suggest that the most straightforward path ahead is for researchers to report HRV metrics in parallel with chronotropic measures (heart period always, HR optionally), to choose the CV adjustment method over other adjustment procedures, and if adjustment is used to always report (untransformed) unadjusted as well as adjusted outcomes.

We also advocate for continued work focused on developing HRV metrics possessing the highest reliability and construct and predictive validity with respect to autonomic control and disease risk. There are both theoretical and empirical avenues to bolster future inferences on autonomic cardiovascular control from HRV metrics. Quantitative simulations and biophysical modeling of the sinoatrial cell function (Dexter, Levy, & Rudy, 1989; Dokos, Celler, & Lovell, 1996; Pyetan, Toledo, Zoran, & Akselrod, 2003; Zhang et al., 2000) are key tools to address open questions about the consequences for various adjustment strategies considered here and those yet to be proposed (see, for instance, https://models.cellml.org/e/fb). The theoretical approach should be mirrored by empirical studies using experimental manipulation of autonomic cardiovascular control to detect effects on adjusted and unadjusted HRV metrics. Pursuing these future avenues should likely not be confined to isolating influences on HRV. Other parameters of cardiovascular physiology (e.g., mean arterial pressure, sensitivity of the baroreflex) exhibit chronotropic dependencies (Abrahamsson, Ahlund, Nordlander, & Lind, 2003; Zaza & Lombardi, 2001) that should similarly be examined.

Finally, adjusted and unadjusted HRV metrics should be subjected to rigorous comparisons as reliable predictors or correlates of behavioral states (e.g., psychological stress), individual differences in symptomatology (e.g., depressive symptoms), and health outcomes (e.g., infarction) that are likely related to vagal activity. The argument “that the higher morbidity and mortality associated with a decrease in HRV is likely to be the result of the concurrent increase in HR” (Monfredi et al., 2014, p. 1342) is empirically falsifiable and can be tested by confirming superior prediction of adjusted metrics over unadjusted metrics, which now still make up the majority of current HRV research.

We thus close by reiterating the famous expression, “If it ain't broke, don't fix it.” In this regard, we do not view metrics of HRV as inherently broken or in need of fixing. Rather, we view HRV metrics as in need of continued empirical and inferential refinement based on firm biological grounding.

8

**Figure 5:**
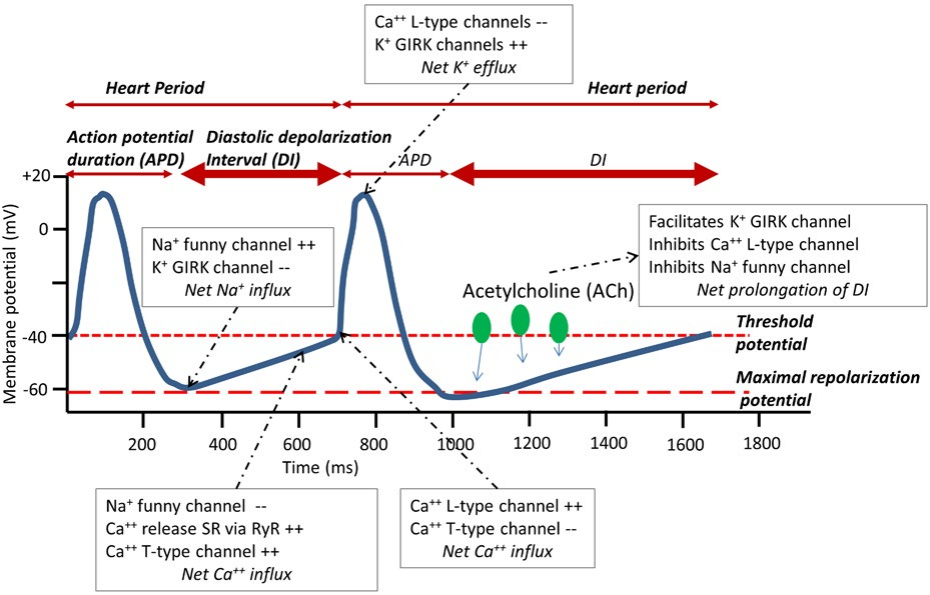
Spontaneous depolarization in the pacemaker cells in the SA node is prolonged by ACh, which, in turn, prolongs the heart period. Main ionic currents related to vagal activity are depicted only; complete rendering would add various sodium currents, the potassium delayed rectifying current, and sodium‐potassium and sodium‐calcium exchangers

**Figure 6:**
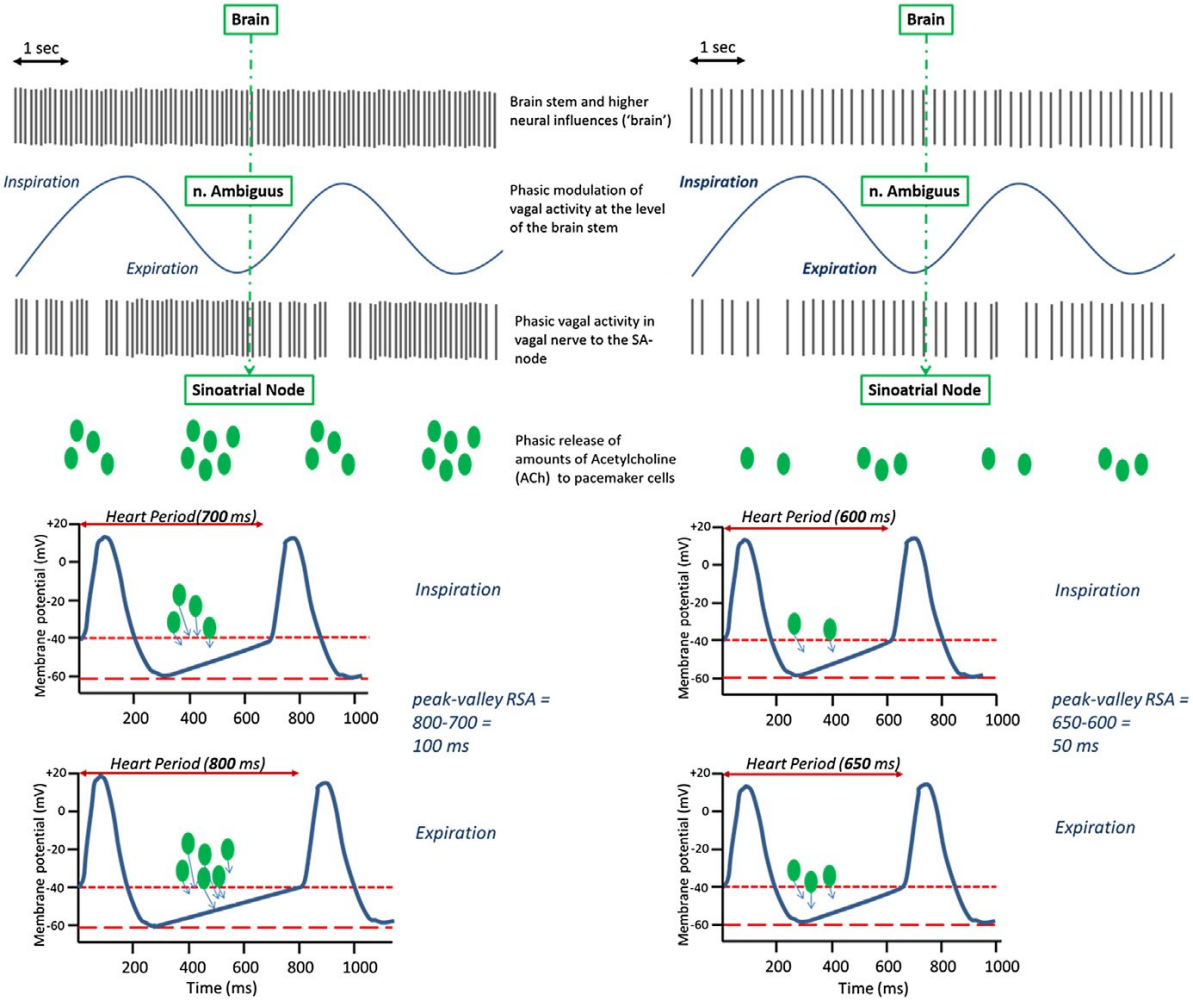
Vagal gating giving rise to respiratory sinus arrhythmia. This is a higher‐order conceptual representation only. In reality, cardiac effector responses to respiration‐related, episodic ACh release do not solely depend on quantity, but also on the timing of its release and clearance, and the ongoing kinetics of the multiple other signal transduction pathways involved in sinoatrial depolarization. (a) High tonic vagal firing (~12 Hz) is reduced during inspiration compared to expiration giving rise to differential amounts of ACh release at the SA effector junction. (b) Gating of lower tonic vagal firing (~6 Hz) will also produce inspiration/expiration differences in the amounts of ACh release, but they are less pronounced as those in (a) where tonic vagal firing is higher

## Notes

1 Two additional metrics can be extracted by spectral analysis, the very‐low (VLF) and low frequency (LF) powers. Because these metrics, like the SDNN, have a less clear autonomic interpretation than RMSSD, HF, and pvRSA (Reyes Del Paso, Langewitz, Mulder, van Roon, & Duschek, 2013), we here focus on RMSSD, HF, and pvRSA, making an exception for SDNN. The latter metric plays a key role in the arguments presented by Monfredi et al. (2014).

2 With the possible exception of the dog, where inspiratory vagal inhibition may be complete or nearly complete (Anrep, Pascual, & Roessler, 1936).
