# Supplementary material for: Should heart rate variability be “corrected” for heart rate? Biological, quantitative, and interpretive considerations
Source: Psychophysiology. 2018 Oct 25;56(2):e13287. doi: 10.1111/psyp.13287 (PMC6378407; doi:10.1111/psyp.13287)
Supplement: Supplementary file 1 [file PSYP-56-na-s001.zip › psyp_13287_Appendix S1.docx]

## An R script created by Michel Nivard & Eco de Geus to run the simulations based on the SEM

## depicted in figure 6 generating the results summarized in table 2 of the paper entitled

## "Should heart rate variability be 'corrected' for heart rate? Biological, quantitative,

## and interpretive considerations"

#install.packages("lavaan")

library(lavaan)

set.seed(1234)

N <- 10000 # rnorm sample size (big-ish)

N_repeat <- 10 # repeat estimation

N_sets <- 10 # number of parameter settings tested

precision <- 0.01 # precision of 0 and 1 in standardisation

# In simulations we can conveniently dictate the content of the core latent variable

# we standardize for easy computations further on

vagal <- rnorm(N,mean=0,sd=1)

# We create BMI dependent on vagal (that explains 10% variance in BMI) and

# choose the residual effects on BMI such that BMI is also on a standardized scale

b_v_bmi <- sqrt(.10) # beta coefficient in BMI = b * vagal + error

error_nv_BMI <- rnorm(N,mean=0,sd=sqrt(1-b_v_bmi^2))

BMI <- b_v_bmi* vagal + error_nv_BMI

# The parameters (b_direct, b_v_ibi, b_v_HRV) can be varied to see the results

# under 10 different parameter settings with different assumptions about the reliability of HRV or IBI

# as indicators of vagal activity and the presence and size of the b_direct effect

# of IBI on HRV. NB: we use only 9 in the paper as the 10^th^ parameter set is considered to be unlikely to

# apply to real IBI and HRV data

# We set up vectors containing the 10 sets:

# setname @ String describing the set's characteristics

# b_direct @ the direct path between IBI and HRV in Figure 6 (can be zero)

# b_v_ibi @ beta coefficient in IBI = b * vagal + error

# b_v_HRV @ beta coefficient in HRV = b * vagal + b_direct * IBI + error

# b_HRV_BMI @ We compute the expected beta's of HRV (b_v_HRV * b_v_bmi + b_direct * b_v_ibi * b_v_bmi)

# b_IBI_BMI @ and IBI on BMI from the SEM in Figure 6 (b_v_ibi * b_v_bmi) using path tracing / covariance rules

# a_sem @ estimated b_v_ibi from SEM on the simulated dataset

# b_sem @ estimated b_v_HRV from SEM on the simulated dataset

# c_sem @ estimated b_direct from SEM on the simulated dataset

# BMIvagal @ beta from regression with lm with one predictore

# BMIvagal_rsq @ explained variance in BMI by vagal

# BMIHRV @ beta from regression with lm with one predictor (HRV)

# BMIHRV_rsq @ explained variance in BMI by HRV

# BMIIBI @ beta from regression with lm with one predictor (IBI)

# BMIIBI_rsq @ explained variance in BMI by IBI

# BMIHRVIBIcov @ beta from regression with lm with HRV as predictor, after regressing out IBI

# BMIHRVIBIcov_rsq @explained variance in BMI by HRV, after regressing out IBI

# BMI_both_IBI @ beta from regression with lm with two predictors (IBI+HRV)

# BMI_both_HRV @ beta from regression with lm with two predictors (IBI+HRV)

# BMI_both_rsq @ explained variance in BMI by IBI and HRV

setname <- c("PARAMSET 1 No direct effect ; IBI and HRV are both as good as indicators of vagal activity",

"PARAMSET 2 No direct effect ; HRV is a better indicator of vagal activity than IBI",

"PARAMSET 3 No direct effect ; IBI is a better indicator of vagal activity than HRV",

"PARAMSET 4 Small direct effect ; IBI and HRV are both as good as indicators of vagal activity",

"PARAMSET 5 Small direct effect ; HRV is a better indicator of vagal activity than IBI",

"PARAMSET 6 Small direct effect ; IBI is a better indicator of vagal activity than HRV",

"PARAMSET 7 Moderate direct effect ; IBI and HRV are both as good as indicators of vagal activity",

"PARAMSET 8 Moderate direct effect ; HRV is a better indicator of vagal activity than IBI",

"PARAMSET 9 Moderate direct effect ; IBI is a better indicator of vagal activity than HRV",

"PARAMSET 10 Figure 4b Moderate direct effect ; only IBI is an indicator of vagal activity")

b_direct <- c(0, 0, 0, 0.1, 0.1, 0.1, 0.3, 0.3, 0.3, 0.3)

b_v_HRV <- c(sqrt(.4), sqrt(.4), sqrt(.1), sqrt(.4), sqrt(.4),sqrt(.1),sqrt(.3),sqrt(.4),sqrt(.1), 0)

b_v_ibi <- c(sqrt(.4), sqrt(.1), sqrt(.4), sqrt(.4), sqrt(.1),sqrt(.4),sqrt(.3),sqrt(.1),sqrt(.4), sqrt(.4))

b_HRV_BMI <- b_v_HRV * b_v_bmi + b_direct * b_v_ibi * b_v_bmi

b_IBI_BMI <- b_v_ibi * b_v_bmi

# and initialize the outcome vectors with zeros

a_sem <- matrix(rep(0,N_sets*N_repeat), nrow = N_sets , ncol = N_repeat)

b_sem <- matrix(rep(0,N_sets*N_repeat), nrow = N_sets , ncol = N_repeat)

c_sem <- matrix(rep(0,N_sets*N_repeat), nrow = N_sets , ncol = N_repeat)

BMIvagal <- matrix(rep(0,N_sets*N_repeat), nrow = N_sets , ncol = N_repeat)

BMIvagal_rsq <- matrix(rep(0,N_sets*N_repeat), nrow = N_sets , ncol = N_repeat)

BMIHRV <- matrix(rep(0,N_sets*N_repeat), nrow = N_sets , ncol = N_repeat)

BMIHRV_rsq <- matrix(rep(0,N_sets*N_repeat), nrow = N_sets , ncol = N_repeat)

BMIIBI<- matrix(rep(0,N_sets*N_repeat), nrow = N_sets , ncol = N_repeat)

BMIIBI_rsq <- matrix(rep(0,N_sets*N_repeat), nrow = N_sets , ncol = N_repeat)

BMIHRVIBIcov <- matrix(rep(0,N_sets*N_repeat), nrow = N_sets , ncol = N_repeat)

BMIHRVIBIcov_rsq <- matrix(rep(0,N_sets*N_repeat), nrow = N_sets , ncol = N_repeat)

BMI_both_IBI <- matrix(rep(0,N_sets*N_repeat), nrow = N_sets , ncol = N_repeat)

BMI_both_HRV <- matrix(rep(0,N_sets*N_repeat), nrow = N_sets , ncol = N_repeat)

BMI_both_rsq <- matrix(rep(0,N_sets*N_repeat), nrow = N_sets , ncol = N_repeat)

# (with apologies to R afficionado’s for the clunky for loops – but they function, so we happily apply)

for (j in 1:N_repeat){

for (i in 1:N_sets){

# We create IBI dependent on vagal and choose the residual effects of e.g. intrinsic HR and SNS

# on IBI such that IBI is on a standardized scale; note that error_IBI collapses the two sources

# of non-vagal variance in Figure 6: that due to non-vagal IBI effects, and all other sources

# of error variance in IBI.

error_IBI <- rnorm(N,mean=0,sd=sqrt(1-b_v_ibi[i]^2))

IBI <- b_v_ibi[i] * vagal + error_IBI

# We create HRV dependent on vagal and (if b_direct >0) IBI. We also choose the residual effects

# of e.g. respiration rate on HRV such that HRV is on a standardized scale

# This is non trivial as vagal and IBI are not independent!

# We first compute the vagal part of the HRV variance falling back on standard

# variance rules: var (aX+bY) = a^2*var(X) + b^2var(Y) + 2abCOV(X,Y)

# with X = vagal, Y = IBI, a = b_v_HRV, and b = b_direct.

# Noting that the variance in vagal and IBI is 1, and that the

# covariance between vagal and IBI (both with variance 1) is the direct path (b_v_ibi[i])

# we can set:

var_HRV_vagal <- b_v_HRV[i]^2 + b_direct[i]^2 + 2 * (b_v_HRV[i] * b_direct[i] * (b_v_ibi[i]))

# we then compute the contribution of the two remaining 'error' sources (bNV_HRV and ENV_HRV in Figure 6)

# so that HRV will also be standardized.

# Because so much has been going on we demand a certain precision by the repeat loop, just to be sure

repeat {

error_HRV <- rnorm(N,mean=0,sd=sqrt(1 - var_HRV_vagal ))

HRV <- b_v_HRV[i] * vagal + b_direct[i] * IBI + error_HRV

if ( (abs( mean (HRV) ) < precision) && (abs( var (HRV) -1 ) < precision) ) break

}

# We recast the four variables into a dataframe with: vagal, IBI, HRV and BMI

dataset <- cbind.data.frame(vagal, IBI, HRV, BMI)

# We now fit the true SEM model as an overall sanity check

# 'a' should be b_v_ibi[i]

# 'b' should be b_v_HRV[i]

# 'c' should be b_direct[i]

model <-'

# outcome model

HRV ~ c*IBI+b*vagal

# mediator models

IBI ~ a*vagal

# indirect effects

IDE := a*b

# total effect

total := c+a*b

'

fitsem <- sem(model,dataset,bootstrap = 10000)

a_sem[i,j] <- coef(fitsem)["a"]

b_sem[i,j] <- coef(fitsem)["b"]

c_sem[i,j] <- coef(fitsem)["c"]

# We fit alternative regression models to predict BMI either from the latent vagal activity

# directly (truth) or by using either IBI or HRV alone as indicators of vagal, or

# by first regressing IBI effects from BMI and using HRV as an IBI-corrected indicator of vagal

# (covariate analysis) or by using both IBI and HRV as indicators of vagal.

# We compare the average estimates

# for the three beta's and the total explained variance in BMI

true_model <- lm(BMI ~ vagal,data=dataset) # BMI predicted by vagal

BMIvagal[i,j] <- summary.lm(true_model)$coefficients[2,1]

BMIvagal_rsq[i,j] <- summary.lm(true_model)$r.squared

hrv_indicator <- lm(BMI ~ HRV,data=dataset) # BMI predicted by HRV

BMIHRV[i,j] <- summary.lm(hrv_indicator)$coefficients[2,1]

BMIHRV_rsq[i,j] <- summary.lm(hrv_indicator)$r.squared

ibi_indicator <- lm(BMI ~ IBI,data=dataset) # BMI predicted by IBI

BMIIBI[i,j] <- summary.lm(ibi_indicator)$coefficients[2,1]

BMIIBI_rsq[i,j] <- summary.lm(ibi_indicator)$r.squared

## The covariate analysis is a bit more involved: we take the residual BMI after regressing

## out IBI as a covariate. We then estimate how much HRV still predicts of this BMI residual

BMI.resid = ibi_indicator$residuals

dataset2 <- cbind(dataset,BMI.resid)

ibi_cov_indicator <- lm(BMI.resid ~ HRV,data=dataset2) # BMIresidual (after IBI) predicted by HRV

BMIHRVIBIcov[i,j] <- summary.lm(ibi_cov_indicator)$coefficients[2,1]

BMIHRVIBIcov_rsq[i,j] <- summary.lm(ibi_cov_indicator)$r.squared

two_indicators <- lm(BMI ~ IBI + HRV,data=dataset) # BMI predicted by IBI and HRV

BMI_both_IBI[i,j] <- summary.lm(two_indicators)$coefficients[2,1]

BMI_both_HRV[i,j] <- summary.lm(two_indicators)$coefficients[3,1]

BMI_both_rsq[i,j] <- summary.lm(two_indicators)$r.squared

rm(true_model,hrv_indicator,ibi_indicator, dataset2, two_indicators)

rm(HRV,IBI) # clean up before next loop

}

}

# collect relevant stuff to generate the output (means across N_repeat)

output <- rep(" ",10)

sink (file="simulate_vagal_IBI_HRV.txt", append=FALSE)

for (i in 1:N_sets){

output[1] <- sprintf("%s\n", setname[i])

output[2] <- sprintf("True b_v_ibi: %5.3f, a recaptured by SEM: %5.3f\n",

b_v_ibi[i],mean(a_sem[i,]))

output[3] <- sprintf("True b_v_HRV: %5.3f, b recaptured by SEM: %5.3f\n",

b_v_HRV[i],mean(b_sem[i,]))

output[4] <- sprintf("True b_direct: %5.3f, c recaptured by SEM: %5.3f\n",

b_direct[i],mean(c_sem[i,]))

output[5] <- sprintf("Vagal predicting BMI (true: %5.3f)\t\tbeta Vagal %5.3f R2: %5.3f\n",

b_v_bmi, mean(BMIvagal[i,]), mean(BMIvagal_rsq[i,]))

output[6] <- sprintf("HRV predicting BMI (true: %5.3f) \t\tbeta HRV %5.3f R2: %5.3f\n",

b_HRV_BMI[i], mean(BMIHRV[i,]), mean(BMIHRV_rsq[i,]))

output[7] <- sprintf("IBI predicting BMI (true: %5.3f) \t\tbeta IBI %5.3f R2: %5.3f\n",

b_IBI_BMI[i], mean(BMIIBI[i,]), mean(BMIIBI_rsq[i,]))

output[8] <- sprintf("HRV predicting residual BMI (after IBI),\tbeta HRV %5.3f R2: %5.3f\n",

mean(BMIHRVIBIcov[i,]), mean(BMIHRVIBIcov_rsq[i,]))

output[9] <- sprintf("HRV+IBI jointly predicting BMI, \t\tbeta HRV %5.3f R2: %5.3f\n",

mean(BMI_both_HRV[i,]), mean(BMI_both_rsq[i,]))

output[10] <- sprintf("HRV+IBI jointly predicting BMI, \t\tbeta IBI %5.3f R2: %5.3f\n",

mean(BMI_both_IBI[i,]),mean(BMI_both_rsq[i,]))

cat(output)

}

sink()
