# Supplementary material for: Should heart rate variability be “corrected” for heart rate? Biological, quantitative, and interpretive considerations
Source: Psychophysiology. 2018 Oct 25;56(2):e13287. doi: 10.1111/psyp.13287 (PMC6378407; doi:10.1111/psyp.13287)
Supplement: Supplementary file 1 [file PSYP-56-na-s001.zip › psyp_13287_figures S1-S6.pptx]

## Slide 1
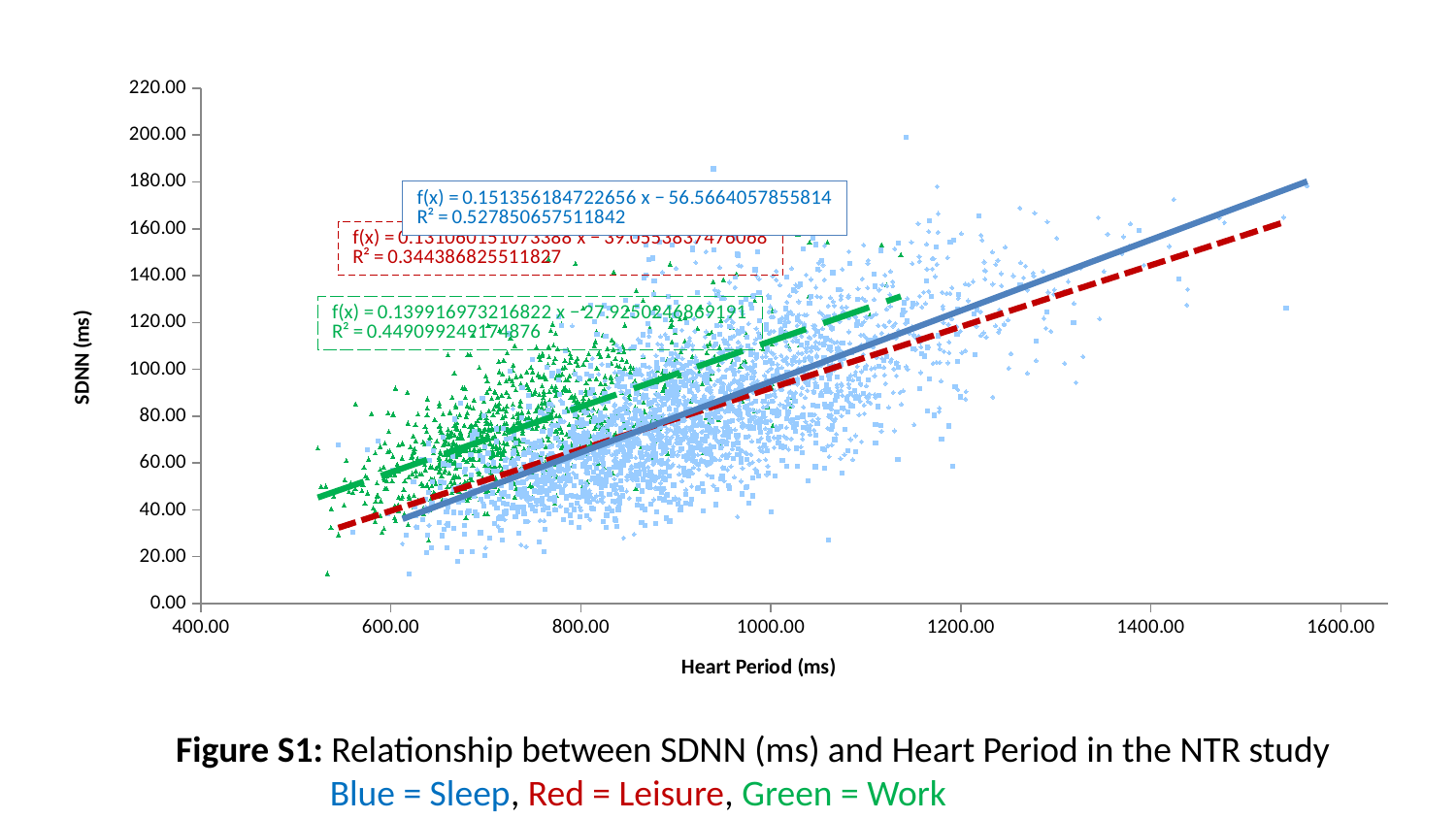

### Chart
| Category | | | |
|---|---|---|---|Figure S1: Relationship between SDNN (ms) and Heart Period in the NTR study
	 Blue = Sleep, Red = Leisure, Green = Work

## Slide 2
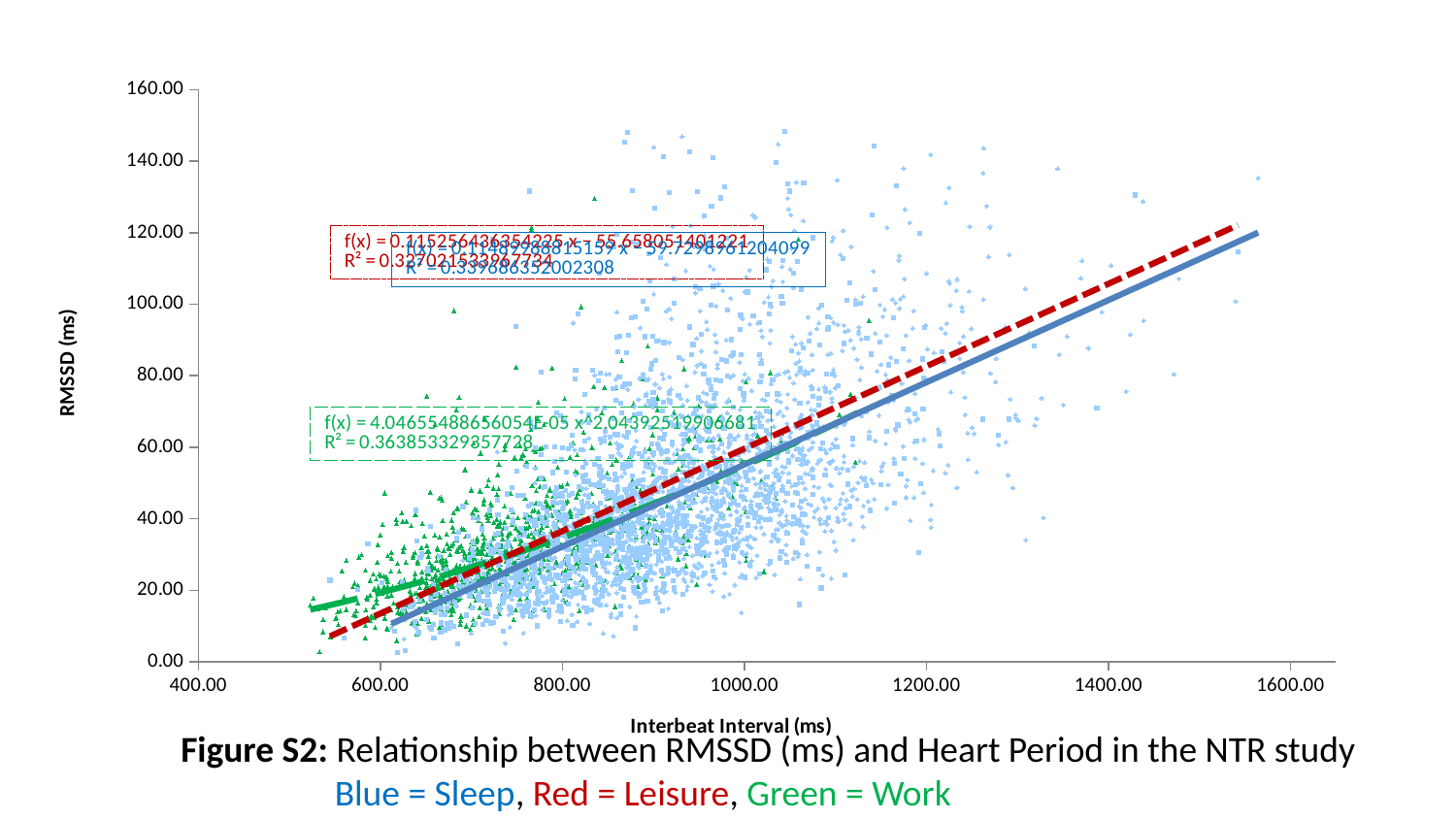

### Chart
| Category | | | |
|---|---|---|---|Figure S2: Relationship between RMSSD (ms) and Heart Period in the NTR study
	 Blue = Sleep, Red = Leisure, Green = Work

## Slide 3
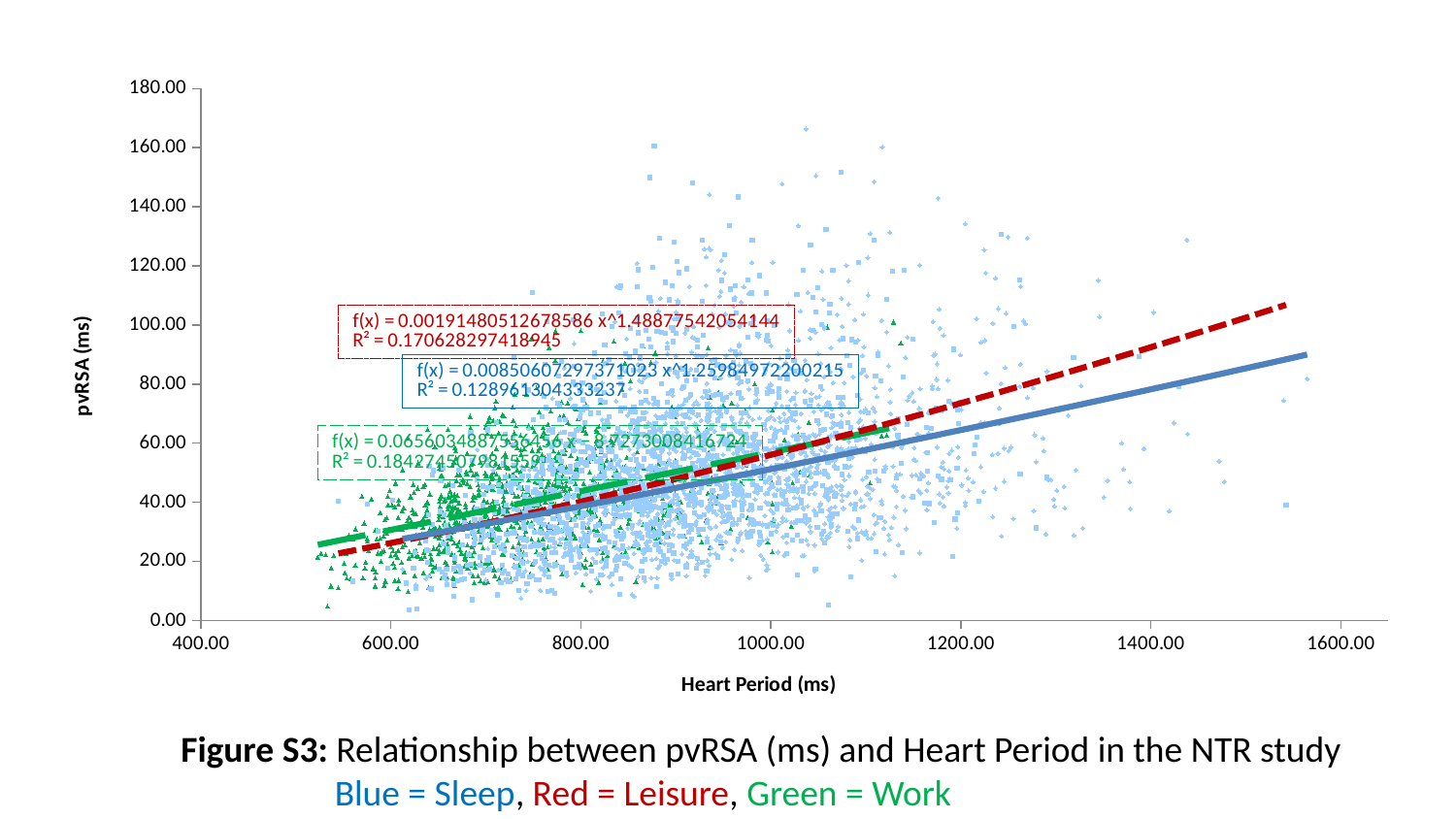

### Chart
| Category | | | |
|---|---|---|---|Figure S3: Relationship between pvRSA (ms) and Heart Period in the NTR study
	 Blue = Sleep, Red = Leisure, Green = Work

## Slide 4
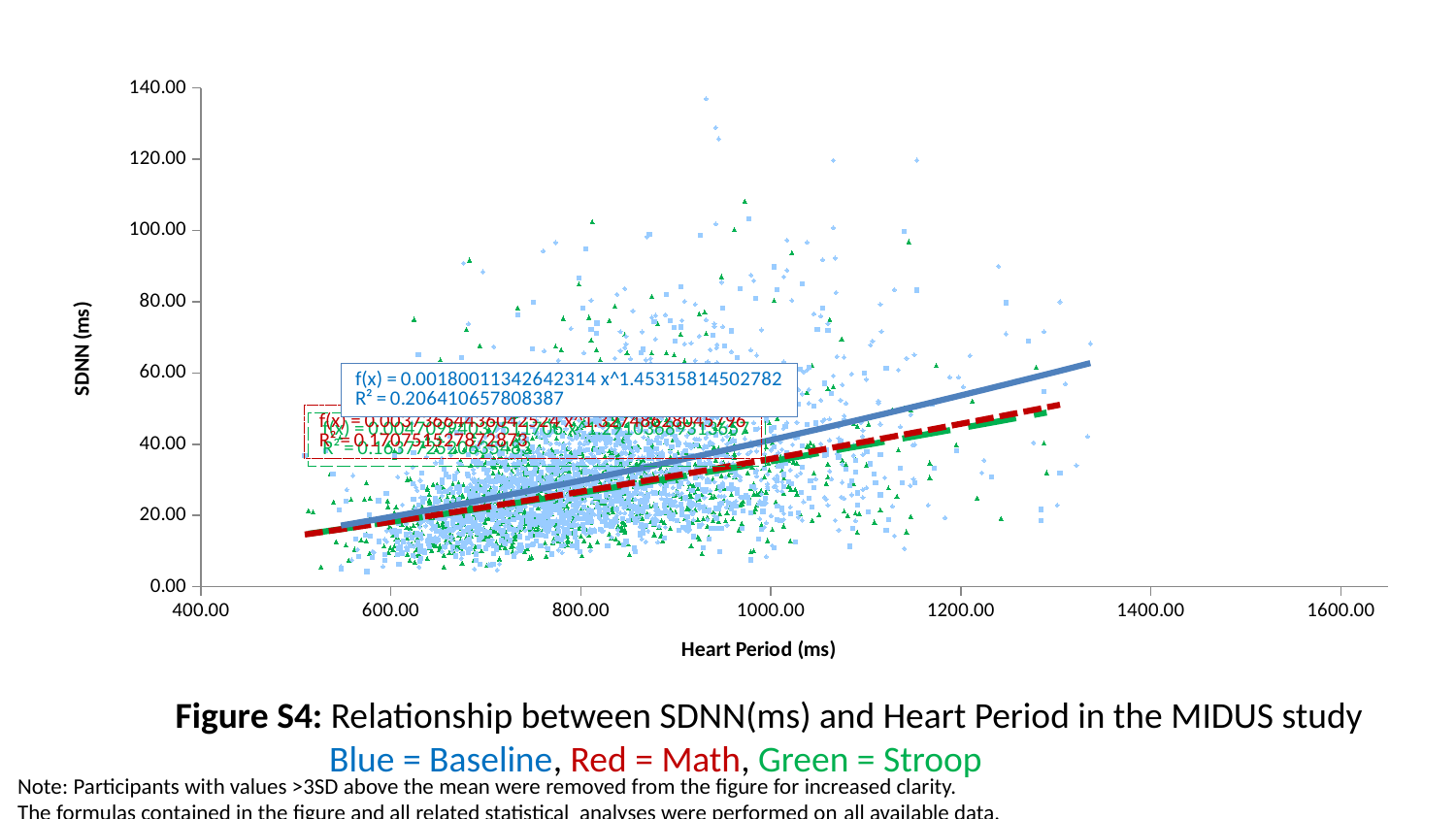

### Chart
| Category | | | |
|---|---|---|---|Figure S4: Relationship between SDNN(ms) and Heart Period in the MIDUS study
	 Blue = Baseline, Red = Math, Green = Stroop
Note: Participants with values >3SD above the mean were removed from the figure for increased clarity.
The formulas contained in the figure and all related statistical analyses were performed on all available data.

## Slide 5
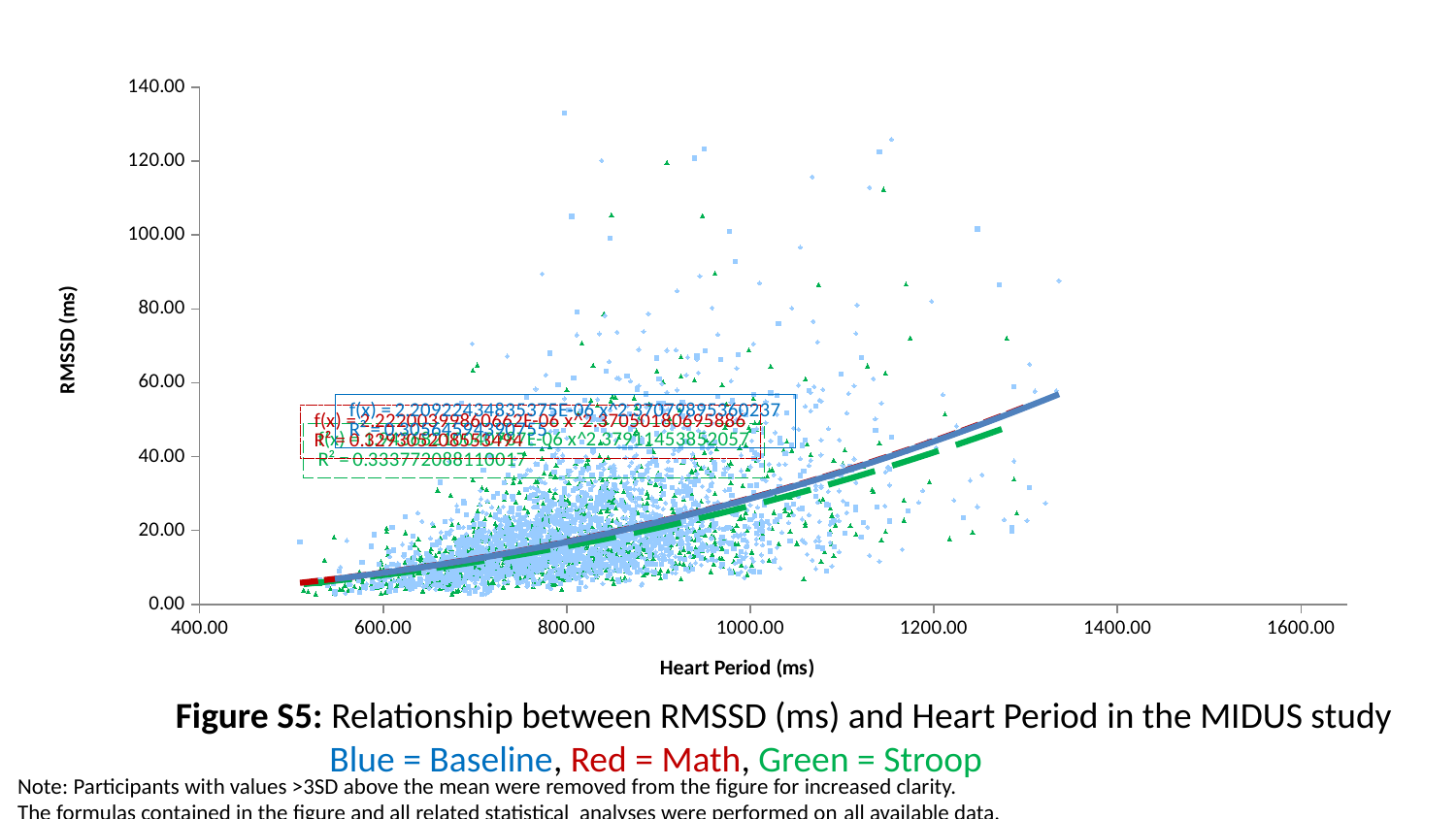

### Chart
| Category | | | |
|---|---|---|---|Figure S5: Relationship between RMSSD (ms) and Heart Period in the MIDUS study
	 Blue = Baseline, Red = Math, Green = Stroop
Note: Participants with values >3SD above the mean were removed from the figure for increased clarity.
The formulas contained in the figure and all related statistical analyses were performed on all available data.

## Slide 6
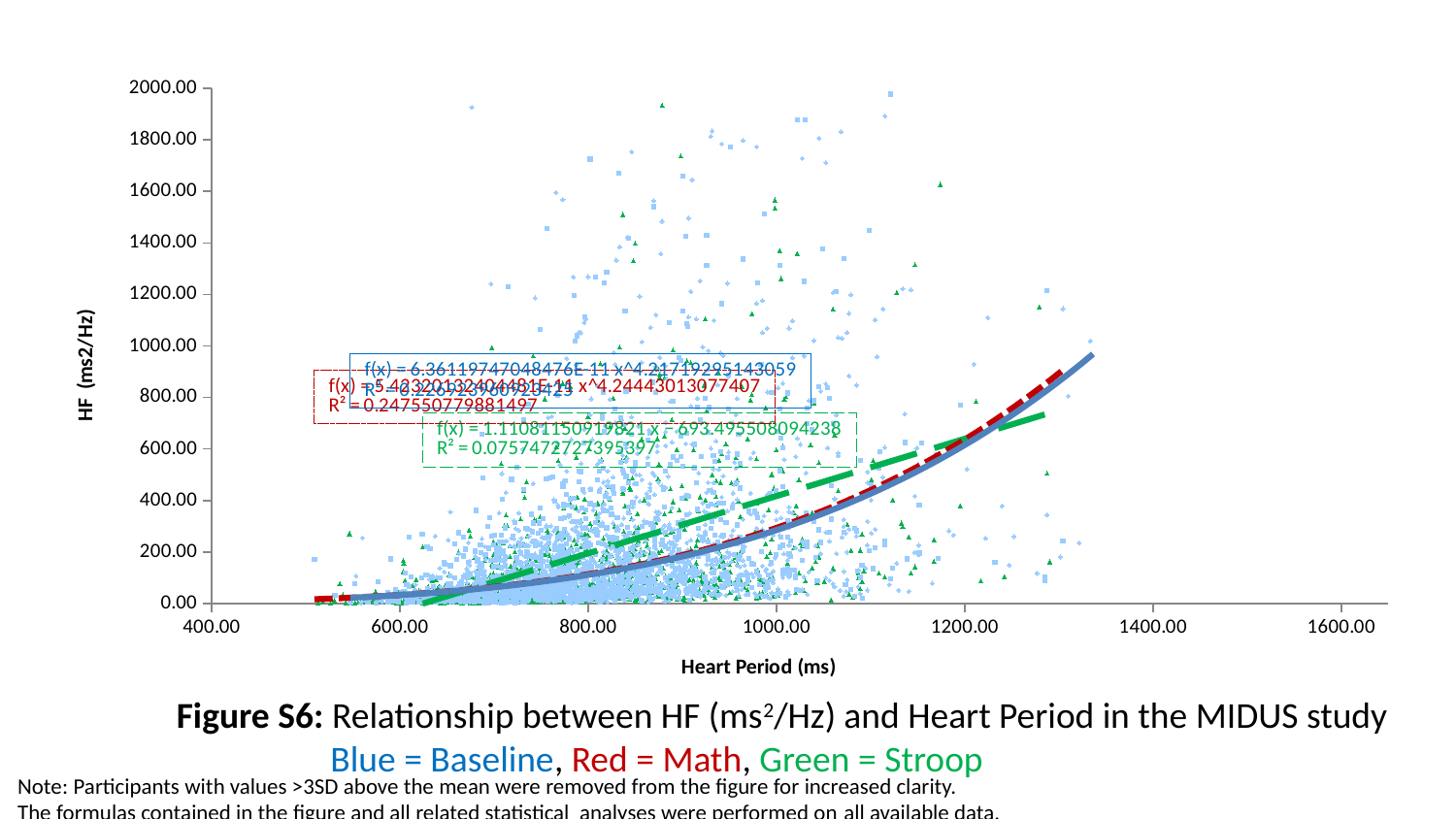

### Chart
| Category | | | |
|---|---|---|---|Figure S6: Relationship between HF (ms2/Hz) and Heart Period in the MIDUS study
	 Blue = Baseline, Red = Math, Green = Stroop
Note: Participants with values >3SD above the mean were removed from the figure for increased clarity.
The formulas contained in the figure and all related statistical analyses were performed on all available data.
